# An overview of systematic reviews on mental health promotion, prevention, and treatment of common mental disorders for refugees, asylum seekers, and internally displaced persons

**DOI:** 10.1002/14651858.CD013458.pub2

**Published:** 2020-09-04

**Authors:** Eleonora Uphoff, Lindsay Robertson, Baltica Cabieses, Francisco J Villalón, Marianna Purgato, Rachel Churchill, Corrado Barbui

**Affiliations:** Cochrane Common Mental DisordersUniversity of YorkYorkUK; Programa de Estudios Sociales en Salud, Instituto de Ciencias e Innovación en Medicina (ICIM), Facultad de Clínica AlemanaUniversidad del DesarrolloSantiagoChile; Department of Neurosciences, Biomedicine and Movement Sciences, Section of PsychiatryUniversity of VeronaVeronaItaly; Centre for Reviews and DisseminationUniversity of YorkYorkUK; Ilusioname FoundationSantiagoChile; Cochrane Global Mental HealthUniversity of VeronaVeronaItaly

## Abstract

**Background:**

Migrants who have been forced to leave their home, such as refugees, asylum seekers, and internally displaced persons (IDP), are likely to experience stressors which may lead to mental health problems. The efficacy of interventions for mental health promotion, prevention, and treatment may differ in this population.

**Objectives:**

With this overview of systematic reviews, we will map the characteristics and methodological quality of existing systematic reviews and registered systematic review protocols on the promotion of mental health and prevention and treatment of common mental disorders among refugees, asylum seekers, and IDPs. The findings from this overview will be used to prioritise and inform future Cochrane reviews on the mental health of involuntary migrants.

**Methods:**

We searched Ovid MEDLINE (1945 onwards), Ovid Embase (1974 onwards), Ovid PsycINFO, ProQuest PTSDpubs, Web of Science Core Collection, Cochrane Database of Systematic Reviews, NIHR Journals Library, CRD databases (archived), DoPHER, Epistemonikos, Health Evidence, 3ie International Initiative for Impact Evaluation, and PROSPERO, to identify systematic reviews of mental health interventions for involuntary migrants. We did not apply any restrictions on date, language, or publication status to the searches. We included systematic reviews or protocols for systematic reviews of interventions aimed at refugees, asylum seekers, and internally displaced persons. Interventions must have been aimed at mental health promotion (for example, classroom‐based well‐being interventions for children), prevention of mental health problems (for example, trauma‐focussed Cognitive Behavioural Therapy to prevent post‐traumatic stress disorder), or treatment of common mental disorders and symptoms (for example, narrative exposure therapy to treat symptoms of trauma). After screening abstracts and full‐text manuscripts in duplicate, we extracted data on the characteristics of the reviews, the interventions examined in reviews, and the number of primary studies included in each review. Methodological quality of the included systematic reviews was assessed using AMSTAR 2.

**Main results:**

The overview includes 23 systematic reviews and 15 registered systematic review protocols.

Of the 23 published systematic reviews, meta‐analyses were conducted in eight reviews. It was more common for the search strategy or inclusion criteria of the reviews to state that studies involving refugees were eligible for inclusion (23/23), than for asylum seekers (14/23) or IDPs (7/23) to be explicitly mentioned. In most reviews, study eligiblity was either not restricted by participant age (9/23), or restricted to adults (10/23). Reviews commonly reported on studies of diagnosis or symptoms of post‐traumatic stress disorder or trauma (11/23) and were less likely to report on depression or anxiety (6/23). In 15 reviews the intervention of interest was focused on/ specific to psychological therapy. Across all 23 reviews, the interventions most commonly identified from primary studies were general Cognitive Behavioural Therapy, Narrative Exposure Therapy, and a range of different integrative and interpersonal therapies. Even though many reviews included studies of participants without a diagnosis of a mental health problem, they often assessed mental health treatments and did not usually distinguish between promotion, prevention, and treatment in the review aims.

Together the 23 systematic reviews included 336 references, of which 175 were unique primary studies. Limitations to the methodological quality of reviews most commonly related to reporting of selection criteria (21/23), absence of a protocol (19/23), reporting of study design (20/23), search strategy (22/23), and funding sources of primary studies (19/23).

**Authors' conclusions:**

Gaps exist in the evidence on mental health interventions for refugees, asylum seekers, and internally displaced persons. Most reviews do not specify that internally displaced persons are included in the selection criteria, even though they make up the majority of involuntary migrants worldwide. Reviews specific to mental health promotion and prevention of common mental disorders are missing, and there is more evidence available for adults or mixed populations than for children. The literature is focused on post‐traumatic stress disorder and trauma‐related symptoms, with less attention for depression and anxiety disorders. Better quality systematic reviews and better report of review design and methods would help those who may use these reviews to inform implementation of mental health interventions.

## Plain language summary

**An overview of systematic reviews on mental health interventions for involuntary migrants**

Refugees, asylum seekers, and internally displaced persons are involuntary migrants, who have often experienced distress when forced to leave their home, on the journey, and in the process of settling in a host country or new environment. Mental health promotion, prevention, and treatments for mental health problems such as depression, anxiety, and post‐traumatic stress disorder may work differently in these groups of people than for the general population. This overview of systematic reviews summarises the characteristics of reviews available on this topic, to help us determine which research questions are the most important to address in future Cochrane reviews.

We searched for systematic reviews and protocols of systematic reviews on mental health promotion, prevention, and treatment of mental health problems for refugees, asylum seekers, and internally displaced persons. Mental health promotion may, for example, involve a classroom‐based well‐being intervention for children. An example of prevention is trauma‐focused therapy to prevent post‐traumatic stress disorder. Treatment may, for example, include psychological therapy for depression. We found 23 systematic reviews and 15 protocols of reviews in progress. Together the 23 published systematic reviews included 336 references, 175 of which were unique studies. Reviews more commonly included refugees and asylum seekers than internally displaced persons, and were more frequently focused on adults than children. There was more attention on the treatment of post‐traumatic stress disorder than there was for mental health promotion or prevention, or for the treatment of depression or anxiety. Studies of Cognitive Behavioural Therapy, Narrative Exposure Therapy, and integrative and interpersonal therapies were most likely to be included in reviews.

The quality of reviews was limited by a range of issues, many of which related to poor reporting of the review methodology.

The evidence available from systematic reviews may not match the need for evidence‐based interventions for the mental health of involuntary migrants. Review authors should consider relevant groups such as internally displaced persons, children, and people with depression or anxiety, and relevant interventions such as those for mental health promotion or prevention, and treatments other than psychological therapy.

## Background

The United Nations estimates there are around 40 million internally displaced persons (IDPs), 25 million refugees, and three million asylum seekers worldwide, and their numbers are growing (UNHCR 2019). While most research on involuntary migrants takes place in high‐income countries, most live in low‐ and middle‐income countries (Wainberg 2017). In addition to experiences in the country of settlement, the circumstances in which people are forced to leave their homes are likely to be extremely stressful and often unsafe. A large priority setting exercise by the World Health Organization (WHO) Global Forum for Health Research identified people exposed to violence or trauma as a top priority for intervention in global mental health (Sharan 2009).

Compared to the general population, migrants who were forced to leave their home are more likely to experience common mental disorders. The efficacy of psychological therapies (talking therapies) may be different in this population. Apart from language and cultural barriers, the availability of treatment and access to treatment may be additionally restricted, depending on the host country. Even in the UK, a high‐income country with a National Health Service, refugees and asylum seekers may, for example, face organisational and logistic as well as cultural, or language barriers to care. For example, the lack of a permanent home address might make it difficult for migrants to register with a general practitioner (GP) and receive notification letters of medical appointments and subsequent results (Fassil 2015).

The Cochrane Global Mental Health Satellite aims to support the production, dissemination, and implementation of systematic reviews relevant to mental health in low‐ and middle‐income countries (Barbui 2017). This includes reviews on the effectiveness of mental health promotion and the prevention and treatment of common mental disorders for refugees, asylum seekers, and internally displaced persons. To ensure that Cochrane Reviews on this topic fill important gaps in the literature, we undertook an overview of systematic reviews, sometimes called a scoping review or review of reviews, to produce a map of the evidence that is currently available. Rather than synthesising data on the effectiveness of interventions from individual studies, in this overview of systematic reviews we describe the characteristics of systematic reviews, published or ongoing (including registered protocols), on mental health promotion, prevention, and treatment of common mental disorders for refugees, asylum seekers, and internally displaced persons. The resulting evidence map highlights the breadth and depth of the evidence and helps to identify priority research questions and inform the development of Cochrane Reviews on this topic.

### Description of the condition

Common mental disorders considered in this review include all depressive and anxiety disorders, including post‐traumatic stress disorder (PTSD). We are furthermore interested in mental health promotion and prevention of these conditions, as well as symptoms of mental health problems without a formal diagnosis.

Major depressive disorder is characterised by a period of at least two weeks of depressed mood, and is nearly always accompanied by a persistent loss of interest or pleasure in activities which were previously considered enjoyable (APA 2013). A range of symptoms may accompany these key features of depression, including weight loss or weight gain, insomnia or hypersomnia, psychomotor agitation or retardation, fatigue, loss of energy, feelings of excessive guilt and worthlessness, diminished concentration, and recurrent thoughts of death (APA 2013). Other depressive disorders listed in the *Diagnostic and Statistical Manual of Mental Disorders*, Fifth Edition (DSM‐5) include those which occur in specific situations (for example, premenstrual dysphoric disorder), disruptive mood dysregulation disorder in children, and persistent depressive disorder (previously also called dysthymia; symptoms last at least two years). Bipolar disorder is not categorised as one of the depressive disorders, although depressive episodes occur as part of bipolar disorder.

Symptoms of depression and anxiety may be present simultaneously (APA 2013). Anxiety disorders, such as generalised anxiety disorder, and trauma‐related disorders, such as PTSD, are treated as separate types of disorders in DSM‐5 (APA 2013). For this review, we considered anxiety disorders (including phobias and panic disorder) and trauma‐ and stressor‐related disorders (reactive attachment disorder, disinhibited social engagement disorder, PTSD, acute stress disorder, and adjustment disorder).

Anxiety disorders share symptoms of excessive fear, worry, and anxiety, and related behavioural changes. Fear is the emotional response to a perceived imminent threat, which may be real or not, whereas anxiety is the anticipation of a threat in the future. Fear is often associated with immediate and quick responses and behaviours, including panic attacks, whereas anxiety is associated with tension, stress, and behaviours of caution and avoidance. Depending on the type of anxiety disorder and varying between patients, other symptoms may include fatigue, restlessness, irritability, difficulty sleeping, and impaired concentration. Generalised anxiety disorder and PTSD may co‐occur (APA 2013).

PTSD can develop after experiencing a traumatic event, or recurring or chronic traumatic experiences. These include experiences or events witnessed first‐hand, as well as contact with others exposed to trauma. PTSD may develop immediately, shortly after the trauma occurs, or more than six months after the traumatic event (delayed‐onset) (APA 2013). PTSD symptoms include: re‐experiencing traumatic events or moments (nightmares, memories, feelings, reactions); avoidance (of people, places, conversations, feelings); hyperarousal (insomnia, irritability, poor concentration); and negative thoughts and feelings (less positive feelings, loss of interest in pleasurable activities, feeling distant from others) (APA 2013).

Even though their circumstances and experiences will differ, all refugees and asylum seekers have left their country of origin because of a well‐founded fear of persecution, conflict, violence, or other dangerous circumstances (UNHCR 2019). All are likely to have experienced adverse circumstances and insecurity in their home country, challenges associated with the migration journey, and challenges upon arrival and through resettlement processes in a new country. A review of Afghan refugees resettled in industrialised countries identified a range of common adverse experiences that impact on mental health, such as witnessing atrocities, losing family members, stressful escape and transit experiences, living in refugee camps, cultural and language barriers, mental health stigma, unemployment, financial hardship, and loss of status, culture, and identity (Alemi 2014).

Studies on prevalence rates of mental illness among migrants, including refugees and asylum seekers, report widely varying estimates. A review of refugees and labour migrants reported pooled prevalence estimates of 44% depression and 40% anxiety among refugees, compared to 20% and 21% respectively among labour migrants (Lindert 2009). A review including 17 studies of adult refugees resettled in Western countries found a prevalence rate of PTSD of around 9% (Fazel 2005). A recent study of 1000 Syrian refugee children and adolescents living in Lebanon and Jordan found that 46% had developed PTSD (Khamis 2019).

People who are internally displaced have been forced to leave their homes over serious safety concerns, and are staying elsewhere but within their country of origin. Prevalence rates of PTSD reported for this group include 54% of adult internally displaced persons in northern Uganda (Roberts 2008), and 56% for people who fled from the aftermath of a tsunami in Sri Lanka (Ranasinghe 2007).

### Description of the interventions

This overview of systematic reviews includes interventions relating to mental health promotion, prevention of common mental disorders, and treatment of common mental disorders.

#### Mental health promotion

Mental health promotion usually targets the entire population (universal), but may target high‐risk populations such as refugees, asylum seekers, and internally displaced persons (selected health promotion). It considers outcomes related to positive aspects of functioning and well‐being rather than ill health, and in this way it is assumed to lower the risk of developing mental disorders (Tol 2015). Mental health promotion interventions include those delivered at an individual level or in a group‐based format. For example, activities to encourage good mental health and development for children may take place in the classroom or in refugee camps. Programmes might be delivered in villages or neighbourhoods, for example in low‐ and middle‐income countries affected by humanitarian crises.

#### Prevention of common mental disorders

Prevention may be universal, selective (focused on vulnerable individuals or groups), or indicated prevention (for those with symptoms but no diagnosis of mental health problems) (Tol 2015). Whereas mental health promotion interventions are likely to encourage good general mental health, prevention can either be focused on general mental health or on specific common mental disorders. Children may receive trauma‐focused cognitive behavioural therapy (CBT) for the prevention of PTSD, which can be delivered in groups in the case of a large‐scale shared trauma (NICE 2018). An often used prevention intervention is single‐session psychologically‐focused debriefing; however, this is not recommended for the prevention of PTSD in adults or children as it may increase rather than decrease the risk of PTSD and depression (Rose 2002).

#### Treatment of common mental disorders

Many interventions aimed at improving symptoms of common mental disorders are available. This overview may identify many different interventions for the treatment of depression, anxiety, and PTSD. We therefore briefly summarise the most commonly used interventions, and courses of treatment recommended by the UK National Institute for Health and Care Excellence (NICE).

##### Cognitive Behavioural Therapy (CBT)

Certain types of CBT may apply specifically to this population, such as Narrative Exposure Therapy (NET), trauma‐focused CBT, stress inoculation therapy or training, and culturally sensitive CBT. Trauma‐focused CBT can be used for those diagnosed with PTSD, or those with PTSD symptoms, while NET is most often used for those with complex or multiple traumas.

##### Other psychotherapy

Therapies for common mental disorders, depending on the severity of symptoms and specific diagnosis, range in intensity from active monitoring, psychoeducation and low‐intensity psychological interventions (relaxation exercises, counselling/non‐directive supportive therapy, self‐help, behavioural activation) to high‐intensity psychological interventions (interpersonal therapy, psychodynamic therapy) (Kendrick 2012). Patients with PTSD may be offered Eye Movement Desensitization and Reprocessing therapy (EMDR). Some argue that arts‐based programmes and expressive and creative therapies (music, drawing, play) may increase accessibility and reduce social stigma among refugee children (McDonald 2017). Creative writing and 'writing for recovery' approaches are used for adults and children in the treatment of PTSD (Baker 2018).

Treatment may be delivered to individuals, couples, or groups. In low‐ and middle‐income countries or settings with limited resources, task‐shifting and multi‐agency collaborative treatments may be more appropriate than one‐to‐one therapy led by highly trained mental health professionals (Silove 2017). Lay counsellors or health workers who have undertaken a short training programme may deliver counselling, behavioural therapy, or social community interventions. Task‐shifting of the delivery of interventions to less specialised workers makes it more feasible to deliver mental health treatments in low‐resource settings, and may increase sustainability of implemented programmes in such settings over time.

###### Transdiagnostic approaches

Over the last few years, experts in global mental health have called for a move away from the traditional system of categorising patients and treatments according to diagnosis, to a more integrated 'transdiagnostic approach' of treatment according to similarity in symptoms. In low‐ and middle‐income countries in particular; this approach may allow for a better use of limited resources in the treatment of patients with a range of symptoms and comorbid mental health conditions (McEvoy 2009). Two examples implemented and evaluated in low‐ and middle‐income countries are Problem Management Plus (Dawson 2015) and the Common Elements Treatment Approach (CETA) (Murray 2014).

##### Medication

Antidepressants might be used for depression and anxiety for children and adolescents when first‐line talking therapies have not worked or in the case of severe symptoms or where talking therapies are not available (NICE 2019). For adults, medication may be indicated, particularly for more severe forms of PTSD, anxiety and depression, and if someone has a preference for drug treatment. For adults with PTSD, antipsychotics may be prescribed to treat disabling psychotic symptoms or psychotic symptoms unresponsive to other treatments in PTSD (NICE 2018).

### How the intervention might work

As the types of interventions identified may vary widely, we describe below the hypothesised working mechanisms of the psychological and pharmacological interventions most commonly used to treat anxiety, depression, and PTSD.

#### Cognitive Behavioural Therapy (CBT)

CBT for depression, anxiety, and PTSD addresses patterns of thought, particularly negative thoughts and beliefs, and aims to change this way of thinking as well as changing behaviours that may accompany negative patterns of thought (Beck 1979).

In NET, a type of CBT, the patient is guided through the construction of an autobiographical narrative, with a focus on traumatic experiences (Schauer 2011). The creation of a coherent, chronological timeline of personal events is thought to help process the traumatic event (Robjant 2010; Schauer 2011). This is a form of exposure therapy, in which a therapist exposes a patient to a traumatic situation, event, or memory. Exposure may be gradual or all at once, and may be aided by images or virtual reality. When a fear is activated by facing it, this fear can then be reprocessed as the patient becomes used to the exposure (habituation), and symptoms are reduced (Foa 2016). Stress inoculation therapy is an example of non‐trauma focused therapy, derived from CBT, and was designed to help people cope with stress.

Trauma‐focused CBT was originally developed for children and adolescents who suffered from sexual abuse and is now used for children, adolescents, and adults (Cohen 2012). It differs from generic CBT in that it is recognises the influence of the child's family, it addresses problems (cognitive, behavioural, somatic, relational) relating to the trauma, and it is adaptable and mindful of family and community values and culture. Components of the intervention, such as engagement with the narrative of the exposure and education on trauma, can be adapted to the age of the patient.

Transdiagnostic CBT is designed for multiple mental disorders, and for people with multiple mental disorders, to target the common elements of multiple and co‐occurring illnesses. It is based on the idea that certain cognitive and behavioural elements are shared across a range of mental health problems and diagnoses (Mansell 2009). Transdiagnostic CBT is both a type of CBT and a 'transdiagnostic approach', but is considered part of the 'CBT' category in this overview.

#### Other psychotherapy

##### Third‐wave CBT and behavioural approaches

Third‐wave CBT approaches differ from the original, traditional model of CBT. They target the individual's relationship with cognitions and emotions, and focus on the function of cognition such as thought suppression or experiential avoidance (an attempt or desire to suppress unwanted internal experiences, such as emotions, thoughts and bodily sensations) (Hofmann 2008). Strategies used to change thinking processes include acceptance and commitment therapy, compassionate mind training, mindfulness‐based therapy, and dialectic behaviour therapy.

Behavioural therapies, for example behavioural activation, seek to achieve change in behavioural patterns and activities rather than cognitive patterns (Kanter 2012).

##### EMDR

EMDR involves treatment in which the therapist instructs the patient to focus on associations with trauma through images, memories, emotions, and thoughts, while simultaneously using visual (rapid eye movements), auditory, or tactile stimuli. This bilateral stimulation is hypothesised to facilitate reprocessing of the disturbing information associated with traumatic memories after which symptoms reduce (Shapiro 2017). There is an ongoing discussion as to whether the bilateral stimulation is an active ingredient of the therapy and a variety of working mechanisms have been proposed. Some argue that relaxation in response to a stimulus in the absence of danger leads to positive mental and physiological changes, while others argue that traumatic images are made less vivid and emotional as the working memory is used for tasks performed simultaneously during EMDR (Landin‐Romero 2018).

##### Social skills and assertiveness

The social interactions in different contexts are the focus of social skills training and assertiveness training for anxiety and depression (Jackson 1985).

##### Psychodynamic therapies

Grounded in psychoanalytic theory (Freud 1949), psychodynamic therapy uses the therapeutic relationship to explore and resolve unconscious conflict through the redirection of emotions to the therapist (transference) and interpretation, with relief of symptoms as an indirect outcome.

##### Creative therapies

Creative therapies may use writing, music, arts, dance/ movement, or drama to recall traumatic memories and process trauma associated with PTSD in a non‐verbal way. Mechanisms of action are thought to include relaxation, activation and expression of memories and emotions, facilitating a sense of control and empowerment resulting from creating art, exposure through symbolic art, and rebuilding of self‐esteem (Baker 2018).

##### Interpersonal, cognitive analytic, humanistic, and other integrative therapies

Humanistic therapies focus on the therapeutic relationship, and therapist values of empathy, genuiness, and unconditional positive regard are hypothesised to facilitate patient insight and change in symptoms (Rogers 1951). Integrative therapies, including counselling, interpersonal therapy, and cognitive analytic therapy, form a group of therapies that combine components of different psychological therapy models, for example from CBT, psychodynamic therapy, and person‐centered approaches (Stiles 2008).

#### Transdiagnostic approaches

Transdiagnostic approaches vary in terms of their key mechanisms of action and may borrow from and combine different treatment approaches. Examples of transdiagnostic approaches implemented in low‐ and middle‐income countries in the last few years include Problem Management Plus and CETA (Common Elements Treatment Approach).

Problem Management Plus combines psychoeducation, motivational interviewing, problem‐solving therapy and behavioural techniques. Problem‐solving therapy and behavioural therapy helps people to manage the day‐to‐day practical problems (such as work, relationships) associated with mental illness. Psychoeducation educates patients both on the effects of adversities on mental health and the rationale of the treatment, while motivational interviewing is used to promote engagement with the treatment (Dawson 2015).

CETA was developed to be delivered by people who are not mental health specialists, in settings with limited resources. Elements of CETA include encouraging engagement with the intervention, psychoeducation on symptoms and the intervention, relaxation strategies, behavioural activation to encourage participation in rewarding activities, coping with emotions, and exposure therapy (Murray 2014). These elements can be delivered in different combinations to address various symptoms.

#### Medication

Antidepressants affect the activity of neurotransmitters such as serotonin and noradrenaline, which in turn is hypothesised to affect the regulation of mood and emotions. Selective Serotonin Reuptake Inhibitors (SSRIs) reduce the reabsorption of serotonin by the brain, which can increase positive feelings. Serotonin and Noradrenaline Reuptake Inhibitors (SNRIs) block the reabsorption of serotonin and noradrenaline. Tricyclic antidepressants (TCAs) are an older class of antidepressants which are no longer commonly used. Most TCAs work by preventing the reuptake of serotonin or noradrenaline, or both (Feighner 1999).

### Why it is important to do this overview

Refugees, asylum seekers, and internally displaced persons are a large and vulnerable group of people, who are more likely than the general population to suffer from a common mental disorder. At present, no Cochrane Review exists on interventions for the promotion of mental health, or the prevention or treatment of common mental disorders in this population. Future Cochrane Reviews may focus on mental health promotion, prevention or treatment, across several common mental disorders, for a wide range of interventions, in different age groups and populations, across different settings. This overview provides an evidence map of systematic reviews conducted on this topic, to identify priority research questions and inform the development of Cochrane Reviews.

## Objectives

To map the characteristics and methodological quality of existing systematic reviews and registered review protocols on the promotion of mental health and prevention and treatment of common mental disorders among refugees, asylum seekers, and internally displaced persons.

Characteristics of interest are:

- the type of systematic review (Cochrane, non‐Cochrane, meta‐analysis, narrative synthesis);
- population (refugees, asylum seekers, internally displaced persons, age, mental health diagnosis);
- setting (country of origin and study setting);
- types of studies (randomised controlled trials, other designs);
- types of interventions (promotion, prevention, treatment; CBT, other psychotherapy, transdiagnostic, medication);
- types of comparators (no treatment, placebo, waiting list, treatment‐as‐usual, other treatment);
- intervention provider (professional, lay health worker);
- review characteristics (included primary studies, review quality).

Whereas a systematic review would normally seek to answer questions related to the effectiveness or efficacy results of included studies, this overview of systematic reviews provides a description of the depth and breadth of the literature available and does not answer questions of effectiveness. Data on study characteristics were extracted to give an overview of systematic reviews, ongoing or published, on this topic.

This overview is part of a Cochrane Global Mental Health satellite project to identify priorities for Cochrane Reviews in global mental health. We will produce an evidence map and a lay summary of literature identified in the overview, to provide a basis to engage with stakeholders within and outside of academia to prioritise Cochrane Reviews of mental health of refugees, asylum seekers and internally displaced persons. This will ensure that the Cochrane Global Mental Health Satellite takes forward research questions seen as a priority by stakeholders to promote a strong evidence base in global mental health.

## Methods

Our overview summarises systematic reviews that include a wide range of participants, interventions, comparators, and outcomes. We followed general principles for conducting an overview of reviews, for example in the search strategy, screening of reviews, and appraisal of the methodological quality of included reviews. Other methods, however, such as the appraisal of primary studies and synthesis of results, are not relevant to the objectives of this overview. The methodology used for this overview therefore also draws on guidance from the Campbell Collaboration on evidence and gap maps (Campbell 2019), methodological guidance published by O’Leary and colleagues (O'Leary 2017), and a review of evidence maps (Miake‐Lye 2016). The protocol is based on the Cochrane systematic review protocol format, as specified in the *Cochrane Handbook for Systematic Reviews of Interventions* (Higgins 2011). Reporting follows PRISMA and PRISMA‐P guidance where applicable (Moher 2009; Shamseer 2015).

### Criteria for considering reviews for inclusion

#### Types of studies

Systematic reviews and protocols of systematic reviews registered in the PROSPERO online database were eligible for inclusion. Reviews had to be clearly identified by the authors as a ‘systematic review’ or ‘meta‐analysis’ in either the title or abstract of the review; and the authors had to present evidence of a systematic search including a search strategy. Cochrane reviews and systematic reviews with and without a meta‐analysis were eligible for inclusion. We included systematic reviews regardless of the study design and methodology of the primary studies. To be included, reviews had to address the evaluation of one or multiple relevant interventions. Systematic reviews were included regardless of the number or breadth of databases searched.

#### Types of participants

We included reviews of studies involving refugees, asylum seekers, and internally displaced persons of all ages. We adopted the following definitions of the UN Refugee Agency (UNHCR), which are derived from the 1951 Convention on the Status of Refugees (UNHCR 2019).

- Refugee: a person who, owing to a well‐founded fear of being persecuted for reasons of race, religion, nationality, membership of a particular social group, or political opinion, is outside the country of his nationality, and is unable to or, owing to such fear, is unwilling to avail himself of the protection of that country.
- Asylum seeker: an individual who is seeking asylum, but whose claim has not yet been finally decided on.
- Internally displaced persons: persons or groups of persons who have been forced or obliged to flee or to leave their homes or places of habitual residence, in particular as a result of, or in order to avoid the effects of, armed conflict, situations of generalised violence, violations of human rights or natural or human‐made disasters, and who have not crossed an internationally recognised border.

Depending on the type of intervention (promotion of mental health, prevention, treatment), participants may have been either diagnosed with depression, anxiety, or PTSD, or experience symptoms associated with one or more of these disorders, or not have any reported symptoms. Although treatment would be expected to be primarily given to participants with a diagnosed common mental disorder, we accepted reviews of treatment interventions with participants without a diagnosis or with elevated symptoms only.

Only systematic reviews including studies with the above population groups were eligible. If samples were mixed, for example including studies conducted in disaster zones and including internally displaced persons, the review was not eligible for inclusion in our overview.

#### Types of interventions

All interventions to promote mental health, or to prevent or treat common mental disorders, were eligible for inclusion. We considered common mental disorders to include anxiety disorders, including PTSD, and depressive disorders, as described in the Description of the condition section above. Eligible interventions included psychotherapies and medication, individual or group treatments, as well as interventions delivered by professionals and lay health workers. We categorised interventions as follows, according to the classification presented in the Description of the interventions section.

- Mental health promotion
- Prevention of common mental disorders
- Treatment of common mental disorders: CBT, other psychotherapy, transdiagnostic approaches, medication.

We planned to adapt this classification if interventions were identified that would not fit any category.

We included only interventions aimed at the promotion of mental health, or the prevention or treatment of common mental disorders, or a combination of these approaches. For example, we excluded reviews of studies evaluating the effects of nutrition or physical activity on mental health outcomes unless the main aim of the intervention was to promote or improve mental health. Interventions were included whether they were targeted at specific groups within the population of refugees, asylum seekers, and internally displaced persons or not, but interventions not aimed at this population were excluded.

We included systematic reviews focussing on general mental health or wellbeing without specifying particular mental health conditions eligible for inclusion.

#### Comparator

All types of comparators were eligible for inclusion. This included any other type of intervention including those part of 'treatment‐as‐usual', no intervention (including waiting list), and any type of placebo.

#### Types of outcome measures

Reviews that reported any mental health‐related outcomes were eligible, irrespective of the measure used or length of follow‐up. This included outcomes relating to symptoms (e.g. severity of anxiety symptoms), diagnosis (e.g. recurrence of depression), functioning, disability, quality of life, and adverse events (for example, hospitalisation or suicidal attempts). Reviews reporting on outcomes relating to constructs of positive psychological constructs, such as wellbeing, were also eligible for inclusion.

### Search methods for identification of reviews

#### Information sources

We searched the following bibliographic databases using key terms relating to the population (refugees, asylum seekers or internally displaced persons; and mental health, including depression, anxiety, PTSD), together with a filter for systematic reviews (Appendix 1).

- Ovid MEDLINE (1946 onwards);
- Ovid Embase (1974 onwards);
- Ovid PsycINFO (all years);
- ProQuest PTSDpubs (all years);
- Web of Science Core Collection (Science and Social Science Indices) (all years).

We supplemented this with a search of the following review databases (all available years).

- Cochrane Database of Systematic Reviews (CDSR)  (www.cochranelibrary.com);
- NIHR Journals Library – Health Technology Assessment (www.journalslibrary.nihr.ac.uk/HTA/#/);
- Centre for Reviews and Dissemination (CRD) Databases (archived) (www.crd.york.ac.uk/crdweb);
- DoPHER (Database of Promoting Health Effectiveness Reviews) (eppi.ioe.ac.uk/webdatabases4/Intro.aspx?ID=9);
- Epistemonikos  (www.epistemonikos.org);
- Health Evidence  (www.healthevidence.org);
- 3ie International Initiative for Impact Evaluation (www.3ieimpact.org/en/evidence/systematic‐reviews/);
- PROSPERO (www.crd.york.ac.uk/prospero).

We checked the reference lists of included systematic reviews to identify additional evidence which may have been missed by the searches.

### Data collection and analysis

We de‐duplicated, uploaded and screened records in Covidence software (Covidence 2020).

#### Selection of reviews

Two review authors (EU, BC) independently screened titles and abstracts against inclusion criteria. We obtained full‐text manuscripts for all titles that were selected during this process, contacting study authors if necessary. Full‐text articles were screened by two review authors (EU, BC) independently, and we resolved disagreements through discussion, with a third review author (RC) to arbitrate if necessary. We recorded reasons for excluding full‐text articles, and present a 'Table: Characteristics of excluded studies'. We collated multiple reports of the same systematic review.

**1:** Excluded studies

**First author**	**Date**	**Reason for exclusion**
Albane	2019	Wrong population
Aly	2017	Wrong population
Demazure	2018	Not a systematic review/systematic review protocol
Esala	2018	Not a systematic review/systematic review protocol
Hassan	2019	Wrong population
Ho	2018	No interventions
Koesters	2018	Not a systematic review/systematic review protocol
Liem	2019	Wrong population
Logan	2018	Wrong population
Murray	2010	Not a systematic review/systematic review protocol
Nicholl	2004	Not a systematic review/systematic review protocol
Purgato	2019	Wrong population
Quosh	2013	Not a systematic review/systematic review protocol
Sijbrandij	2018	Not a systematic review/systematic review protocol
Slobodin	2015	Wrong population
Slobodin	2015a	Not a systematic review/systematic review protocol
Sullivan	2016	Wrong population
Wood	2018	Not a systematic review/systematic review protocol

We based selection of reviews on the inclusion and exclusion criteria relating to types of studies, participants, and interventions. We included systematic reviews regardless of reported outcomes, date and language of publication, and study quality.

For the NIHR Journals Library, PROSPERO, and Epistemonikos, abstracts could not be downloaded and imported in to Covidence. Results from these searches were screened by one reviewer (EU) on the website and, if relevant, records were added to Covidence for full‐text screening in duplicate (see Differences between protocol and review).

#### Data extraction and management

We created a data extraction sheet in Microsoft Excel to collect data from included systematic reviews, and two authors piloted the data extraction sheet by entering data from the first three included systematic reviews, making adjustments if necessary.

We recorded the following information:

- Publication information: first author, year of publication, research group;
- Type of review: Cochrane or non‐Cochrane, published protocol (yes/no), meta‐analyses (yes/no);
- Population of interest at review level: involuntary migrant population (refugees, asylum seekers, internally displaced person), age (adult/child/mix), mental health diagnosis (PTSD, anxiety, depression, mix, other);
- Countries of primary studies eligible for inclusion in the review: included countries/regions of origin, included study settings;
- Intervention type (psychological, pharmaceutical, other) eligible for inclusion in the review, specific interventions identified in the review (see Description of the interventions);
- Comparators eligible for inclusion at review level (no treatment, placebo, waiting list, treatment‐as‐usual, other treatment);
- Intervention provider at review level (professional, lay worker, mix, other including non‐specialist from a non‐governmental organisation);
- Types of primary studies (RCTs, other designs);
- References of included primary studies.

A separate spreadsheet was used to record the methodological quality of the included systematic reviews. For protocols of systematic reviews not yet completed, we collected as much of this information as possible.

We developed a guidance document with an explanation of each of the data extraction items and their categories for all authors taking part in data extraction, to ensure that authors extracted the same data using the same categories for the different variables of interest.

We did not extract data on the effects of interventions from the included systematic reviews or their included primary studies, because the aim of the overview was to map review characteristics.

We planned to extract references of the included primary studies from each systematic review so that we could create a matrix of primary studies in included systematic reviews or order to assess overlap in the primary studies reported by various reviews. We did not plan to extract information from the primary studies, because for this overview we were interested in characteristics of the reviews rather than the primary studies included in the reviews.

Data extraction was performed in duplicate by two reviewers (EU, BC, LR, and FVJ), and we resolved any disagreements through discussion, with the involvement of a third author if required (MP, CB, RC).

Since this overview does not report outcome data, we did not include a 'Summary of findings' table.

#### Assessment of methodological quality of included reviews

We used AMSTAR 2 to critically appraise included systematic reviews (Shea 2017). This tool is suitable for reviews including randomised and non‐randomised studies. It includes 16 domains relating to the research question, review design, search strategy, study selection, data extraction, justification for excluded studies, description of included studies, risk of bias, sources of funding, meta‐analysis, heterogeneity, publication bias, and conflicts of interest.

AMSTAR guidance for the following domains is particularly relevant to the interpretation of our assessment findings.

- Literature searches: A comprehensive search strategy includes a search conducted within 24 months of completing the review, expert consultation, and searching of reference lists of included studies, trial registries, and grey literature if relevant.
- Study selection: Screening and selection of studies should be performed in duplicate, or with a sample performed in duplicate with good agreement between the reviewers.
- Data extraction: Data extraction should be performed in duplicate by two reviewers, or with a sample performed in duplicate and good agreement between reviewers.
- Meta‐analysis: If no meta‐analyses are performed, authors are still expected to discuss any potential heterogeneity in the results and how this may affect the conclusions of the review.

For many of the AMSTAR 2 domains, a positive response is only possible if the required information is reported in the review paper or protocol. The quality rating of the design and conduct of a review therefore depends heavily on the quality of the reporting of a review. We contacted authors in case of missing information and used this information to inform the quality assessment. For protocols of reviews not yet completed, we did not perform a quality assessment.

We used findings from the AMSTAR 2 critical appraisal to understand the certainty of the evidence base of systematic reviews, which in turn informs what future systematic reviews and primary research is needed.

Our approach deviates from the AMSTAR 2 guidance as we only used the individual domains and we did not produce ratings of overall confidence in the findings of each review. Since our overview did not extract data on the findings of reviews, we did not deem ratings in the confidence of review findings appropriate.

In the discussion section of this review, we specifically consider the characteristics of two high‐quality systematic reviews included in our overview. We did not prespecify criteria for high quality reviews. The two highlighted reviews received the highest quality assessment rating for the majority of AMSTAR 2 domains (see Differences between protocol and review).

#### Data synthesis

We reported results as a narrative synthesis of the characteristics of included systematic reviews.

We included the following.

- A table of all characteristics of included systematic reviews specified in the 'Data extraction and management' section.
- A description of ongoing reviews with study characteristics based on registered or published review protocols.
- An inventory of all interventions and comparators included in the identified systematic reviews.
- An assessment of overlap in primary studies included in the selected reviews.
- A figure of the evidence and gaps in the evidence.

## Results

Searches were conducted on the 4th of September 2019. Two authors (EU and BC) screened titles and abstracts of 4613 records and 63 were included for full‐text screening (EU, BC) (Figure). The most common reasons for exclusion were that the review included the wrong population (N = 8), for example, migrants instead of refugees, asylum seekers, and internally displaced persons only, and that the review was not a systematic review or systematic review protocol (N = 8). All excluded studies with reasons for exclusion are listed in the excluded studies table (Table). For three studies, we could not include findings because no full text could be obtained (Anders 2016; Khan 2018; Piegenschke 2019). Reviews and protocols were published in English, except for two reviews in German (Anders 2016; Piegenschke 2019), and one review in Chinese (Liu 2009a).

**1:**
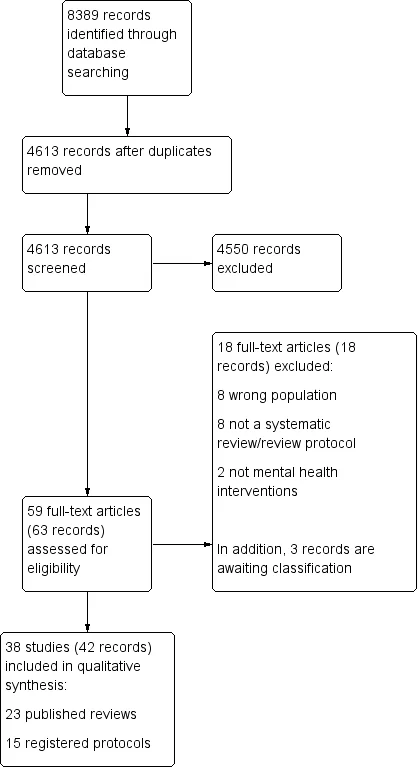
Study flow diagram.

### Description of included reviews

We included 23 published systematic reviews including one review of systematic reviews, and 15 protocols of additional ongoing or planned systematic reviews registered in PROSPERO (https://www.crd.york.ac.uk/prospero/). In this section we first describe the registered protocols and then the published reviews.

#### Registered protocols of ongoing systematic reviews

We found fifteen protocols of systematic reviews registered in PROSPERO, which were planned or still ongoing. One of these is an unpublished Cochrane review protocol in progress (Soltan 2018). Twelve protocols specified plans to conduct meta‐analyses; seven of these included only RCTs (Alzaghoul 2019; Jaroudy 2018; Lindert 2016; Miyazaki 2018; Nosè 2016; Turrini 2019a; Wright 2019).

In this section, we summarise the review questions and eligibility criteria of registered review protocols. If protocols did not specify certain aspects of inclusion and exclusion criteria, we assumed these criteria were not used to select studies. For example, if trial setting was not specified, we assumed trials conducted in any setting were included.

An overview of all included protocols can be found in Table.

**2:** Registered protocols of planned/ongoing systematic reviews

**Author**	**Date of registration**	**Status**	***Scope and selection criteria***					
			**Region/ country of origin**	**Study setting**	**Study design**	**Population**	**Intervention**	**Comparator**
Alzaghoul 2019	Jul‐19	unknown	Low‐income countries Middle East	Middle East low‐income countries	RCTs^1^	children and adult refugees and displaced people with PTSD^2^	any	any except pharmacological
Aslam 2018	Aug‐18	to be published	any	any	quantitative and qualitative	refugees and asylum seekers of any age without diagnosis	community‐based psychological interventions delivered by lay workers	no intervention, usual care, or care limited to information provision or signposting
Danmole 2017	Jun‐17	not published	any	Europe	RCTs, cohort studies, case‐control studies published after 2000	refugees and asylum seekers of any age without diagnosis	all preventative interventions	any
Hameed 2017	May‐17	will not be completed	any	any	any	refugees and asylum seekers of any age with PTSD	medication, psychological therapy, social interventions	no intervention, treatment‐as‐usual, or waiting list
Jaroudy 2018	Jul‐18	unknown	any	low‐ and middle‐income countries	RCTs	adult refugees or IDPs^3^ who have been exposed to conflict with anxiety, PTSD, or depression	any	any
Kobayashi 2018	Jan‐18	ongoing	any	any	unclear	refugees, asylum seekers, and IDPs of any age	(Participatory) Action Research	any
Lawton 2019	Jan‐19	to be published	any	any	any	child asylum seekers and refugees with PTSD, depression, anxiety, or psychological distress	CBT^4^	any
Lindert 2016	Jan‐16	ongoing	any	any	RCTs	asylum seekers and refugees	psychological	any
Meinhart 2017	Jun‐17	unknown	Syria	any	any	adult refugees	any	any
Miyazaki 2018	Jan‐18	unknown	any	any	RCTs	internally and internationally displaced children with any mental health diagnosis or symptoms	low‐intensity psychological therapies	no treatment, treatment‐as‐usual, standard care, same class of therapy
Nosè 2016	Oct‐16	ongoing	any	any	RCTs	asylum seekers and refugees with trauma‐related disorders including PTSD	NET^5^	any
Phillips 2017	Mar‐17	to be published	any	any	quantitative studies with comparator group and qualitative studies	asylum seekers and refugees with any mental health symptoms or diagnosis	any visual or tactile arts‐based therapy	any
Soltan 2018	Jun‐18	ongoing	any	high‐income countries	RCTs for effectiveness	children and adolescent refugees and asylum seekers with any mental health symptoms or diagnosis	community‐based intervention	any
Turrini 2019a	Mar‐19	ongoing	any	any	RCTs	adult asylum seekers and refugees with PTSD	any psychological or social or rehabilitation intervention	any
Wright 2019	Feb‐19	to be published	any	high‐income countries	RCTs	adult refugees and asylum seekers with trauma	NET	any

CBT = cognitive behavioural therapy IDP = internally displaced person NET = narrative exposure therapy PTSD = post‐traumatic stress disorder RCT = Randomised Controlled Trial

##### Scope and selection criteria

Most of the protocols stated that primary studies in any setting would be eligible for inclusion (10/15). One review planned to include studies from Europe (Danmole 2017), two planned to include studies conducted in high‐income countries (Soltan 2018; Wright 2019), one to include studies from low‐ and middle‐income countries (Jaroudy 2018), and according to one protocol studies from low‐income countries in the Middle East were eligible for inclusion (Alzaghoul 2019).

###### Study participants

All fifteen review protocols specified that primary studies of refugees were eligible for inclusion. Twelve specified that asylum seekers were included, and four reviews listed internally displaced persons as participants eligible for inclusion (Alzaghoul 2019; Jaroudy 2018; Kobayashi 2018; Miyazaki 2018). Protocols included participants of all ages (8/15), children or children and adolescents only (Lawton 2019; Miyazaki 2018; Soltan 2018), or adults only (Jaroudy 2018; Meinhart 2017; Turrini 2019a; Wright 2019).

Some of the review protocols focussed on participants with a specific diagnosis, such as PTSD or trauma‐related illness (5/15), or a range of diagnoses, such as depression, anxiety, and PTSD (Jaroudy 2018; Lawton 2019). Other review protocols included any mental health problem or diagnosis (4/15) or measured mental health outcomes in participants who did not necessarily have a mental health condition (4/15).

###### Interventions and comparators

While in some review protocols any interventions were eligible for inclusion (4/15), others focused on specific or several types of therapy (NET, CBT, arts‐based, low‐intensity) (6/15), or a broad range of interventions (3/15). One review protocol focused on preventative interventions, and included only community‐based interventions (Soltan 2018). Another review protocol was of community‐based interventions delivered by lay workers (Aslam 2018). All other review protocols did not specify the intervention provider eligible for inclusion.

For most review protocols, any comparator was eligible for inclusion (11/15), while for four review protocols eligible comparators were specified. One review protocol included any comparator except pharmacological treatment (Alzaghoul 2019); the other three included a range of comparators such as no intervention, treatment‐as‐usual, waiting list, or other therapy (Aslam 2018; Hameed 2017; Miyazaki 2018).

##### Review status

In November 2019, we contacted authors of all review protocols registered in PROSPERO to enquire about the status of the review. Three authors did not respond (Danmole 2017; Jaroudy 2018; Miyazaki 2018). The author of one protocol indicated that their review would not be completed or published (Hameed 2017). Five reviews were ongoing at the time of our enquiry and five reviews were either nearing submission for publication or under review with a journal.

#### Completed (published) systematic reviews

None of the published systematic reviews were Cochrane reviews. All were published in the last ten years, between 2009 and 2019. Meta‐analyses were conducted in eight reviews, four of which included only RCTs (Crumlish 2010; Lambert 2015; Morina 2019; Turrini 2017). Published reviews included in this overview are summarised in Table.

**3:** Included systematic reviews

**Author**	**Date**	**Meta‐analyses**	***Scope and selection criteria***						**No. included studies**
			**Region/country of origin**	**Study setting**	**Study design**	**Population**	**Interventions**	**Comparators**	
Alfadhli 2016	2016	N^1^	developing countries	any	unclear	Refugees of conflict and IDPs^4^	Psychological support	any	60
Crumlish 2010	2010	N	any	any	RCTs^3^	Refugees, asylum seekers, and IDPs with PTSD^5^	pharmacological and psychological	placebo or active comparator (pharmacological), any (psychological)	10
Eberle‐Sejari 2015	2015	Y^2^	any	any	primary research with ≥5 participants	Child refugees, asylum seekers, and IDPs with PTSD	any treatment	any	10
Gwozdziewycz 2013	2013	Y	any	any	quantitative	Refugees who experienced trauma	NET	any	7
Lambert 2015	2015	Y	any	any	RCTs	Adult refugees who experienced, trauma or have PTSD or depression	psychological	any	12
Liu 2009a	2009	N	any	any	primary research	Involuntary migrants	any prevention and treatment	any	35
Mitra 2019	2019	N	any	any	observational	Unaccompanied child refugees and asylum seekers	psychotherapeutic	any	4
Morina 2019	2017	Y	any	any	RCTs with ≥ 10 participants	Refugees and IDPs with PTSD or depression (any age but only findings for children reported)	psychological	any	8
Nakeyar 2016	2016	N	Syria, Iraq, Iran (Kurdish)	developed countries	primary research or literature review published from 2011	Refugees with PTSD	psychological	any	2
Naseh 2019	2019	N	any	any	RCTs	Adult refugees with PTSD	psychological	any	11
Nickerson 2011	2011	N	any	any	any except case studies	Adult refugees and asylum seekers with PTSD	psychological	any	19
Nocon 2017	2017	Y	any	any	primary research	Child refugees and IDPs with trauma‐related disorders	any	any	23
Nosè 2017	2017	Y	any	high‐income countries	RCTs and controlled clinical trials	Adult refugees and asylum seekers with PTSD	psychological	any except psychological intervention	14
Palic 2011	2011	N	any	any	RCTs, controlled clinical trials, pre‐post studies	Tortured or traumatised adult refugees, asylum seekers, and IDPs with PTSD, anxiety, or depression	psychological	any	25
Sims 2017	2017	N	any	any	any	Adult refugees and asylum seekers who experienced trauma	any	any	3
Sonne 2017	2017	N	any	any	any	Adult refugees with PTSD or depression	pharmacological	any	15
Thompson 2018	2018	Y	any	any	RCTs	Adult refugees and asylum seekers with PTSD	psychological	any	16
Tribe 2019	2017	N	any	any	any except case studies and < 10 participants	Traumatised adult refugees and asylum seekers with PTSD, depression, or anxiety	psychological	any	40
Turrini 2017	2017	Y	any	any	RCTs	Refugees and asylum seekers with diagnosis or symptoms of PTSD, depression, or anxiety	any psychological or social or rehabilitation intervention	any	26
Turrini 2019	2019	N	any	any	systematic reviews	Refugees and asylum seekers with a mental health disorder	psychological and pharmacological	any	14
Tyrer 2014	2014	N	any	any	all controlled studies	Child and adolescent refugees, asylum seekers, and IDPs	community‐based	any	21
Van Wyk 2014	2014	N	any	any	any except clinical trials and > 10 participants	Adult refugees and asylum seekers	any	none	7
Williams 2011	2011	N	any	any	any	Refugees	community‐based	any	14

IDP = internally displaced person N = no PTSD = post‐traumatic stress disorder RCT = Randomised Controlled Trial Y = yes

##### Scope and selection criteria

Reviews mostly included studies from any setting. Two reviews included only studies from high‐income countries (Nosè 2017) or developed countries (Nakeyar 2016).

###### Study participants

Refugees were explicitly included in the search strategy or selection criteria of all reviews, asylum seekers in 14 out of 23 reviews, and internally displaced persons in seven out of 23 reviews. Reviews included study participants of any age (9/23), children or children and adolescents only (4/23), or adults only (10/23). Reviews most commonly focused on a diagnosis or symptoms of PTSD or trauma (11/23). Others included various disorders such as PTSD, anxiety, and depression (5/23), or measured mental health in general or included any mental health problem (7/23). Figure shows the number of systematic reviews identified by type of mental health condition, participant age group, and category of refugee.

**2:**
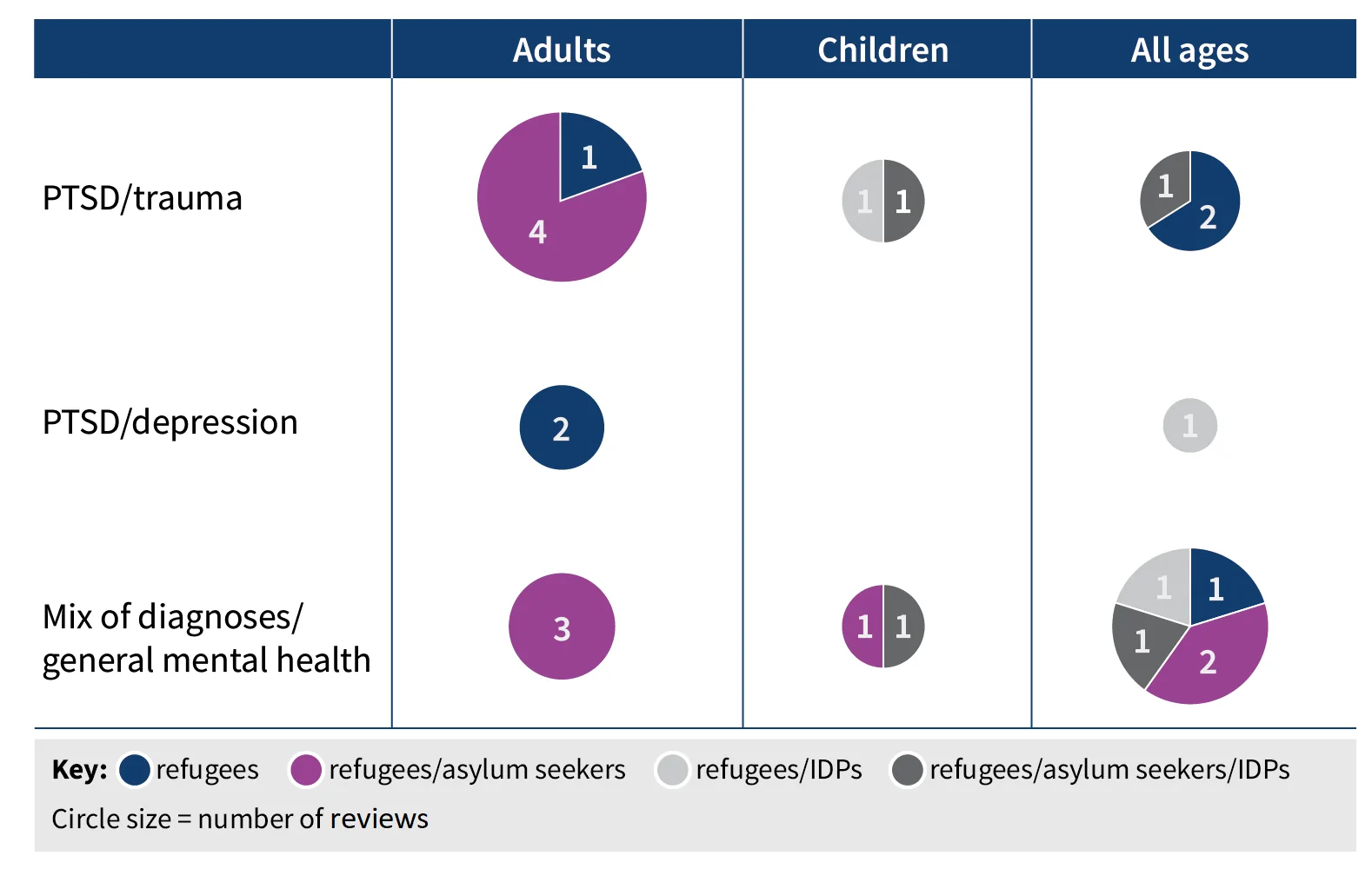
Evidence and gap map

###### Interventions and comparators

Most reviews were focussed on psychological interventions, sometimes called psychosocial interventions by review authors, or psychotherapy (15/23), and two of these reviews also included pharmacological treatments (Crumlish 2010; Turrini 2019). One review included only studies of pharmacological interventions (Sonne 2017), two focused on community‐based interventions (Tyrer 2014; Williams 2011), and five included any intervention (Eberle‐Sejari 2015; Liu 2009a; Nocon 2017; Sims 2017; Van Wyk 2014). Although many reviews included studies of participants who had not been diagnosed with a mental health condition, none of these focused on mental health promotion or prevention interventions. Given the nature of the interventions, it appeared these reviews focussed on treatment of mental health conditions or symptoms rather than prevention.

Most reviews included any type of comparator (21/23). One considered active comparators or placebo for pharmacological interventions, and any comparator for psychological interventions (Crumlish 2010). One review did not include any comparators as it was a review of studies without a control group (Van Wyk 2014).

##### Included studies within completed systematic reviews

###### Number of included studies and unique studies

In total, the 23 systematic reviews included in this overview comprised 336 references to primary studies, of which 175 were unique primary studies (see Appendix 2). A large number of studies was included in only one of the systematic reviews (N = 113), while the two most frequently included RCTs were included in nine systematic reviews (Neuner 2008; Neuner 2010).

###### Interventions and comparators identified

Included reviews largely focused on interventions designed to treat patients with a common mental disorder, or to treat symptoms of a mental health problem. Only one review explicitly included interventions aimed at preventing common mental disorders (Liu 2009a).

Table shows categories of interventions identified in reviews. Across all 23 reviews, the most commonly identified interventions were CBT approaches including general CBT (15/23), NET (17/23), and trauma‐focused CBT (6/23), third‐wave CBT and behavioural approaches (5/23), integrative and interpersonal therapies (30/23), trauma therapies including EMDR (9/23), other trauma‐focused therapy (5/23), and testimony therapy (5/23), transdiagnostic therapy (2/23), psychodynamic therapy (5/23), creative therapies (8/23), education (3/23), medication (4/23), and medication in combination with psychological therapy (1/23).

**4:** Interventions in primary studies

**Type of intervention**	**Specific intervention**	**Number of reviews**
CBT	CBT^1^ (unspecified/general)	15
	NET^2^ (including KIDNET^3^)	17
	Trauma‐focused CBT	6
Third‐wave CBT and behavioural approaches	Stress Inoculation Training	2
	Cognitive Processing Therapy	1
	Biofeedback‐based CBT	1
	Behavioural therapy	1
Integrative and interpersonal therapies	Interpersonal therapy	7
	Counseling	6
	Multimodal therapy	6
	Bespoke/unspecified therapy	11
Trauma therapies	EMDR^4^	9
	Trauma‐focused therapy	5
	Testimony therapy	5
Transdiagnostic therapy (CETA^5^)		2
Psychodynamic therapy		5
Creative therapy (arts, Writing for Recovery, play‐based)		8
Education (of parents, patients, teachers)		3
Medication	Medication alone	4
	Medication in combination with psychotherapy	1

CBT = cognitive behavioural therapy CETA = Common Elements Treatment Approach EMDR = Eye Movement Desensitization and Reprocessing KIDNET = narrative exposure therapy for children NET = narrative exposure therapy

### Methodological quality of included reviews

We assessed the methodological quality of all included systematic reviews in duplicate using AMSTAR 2. Assessments of all reviews per AMSTAR 2 domain are shown in Figure.

**3:**
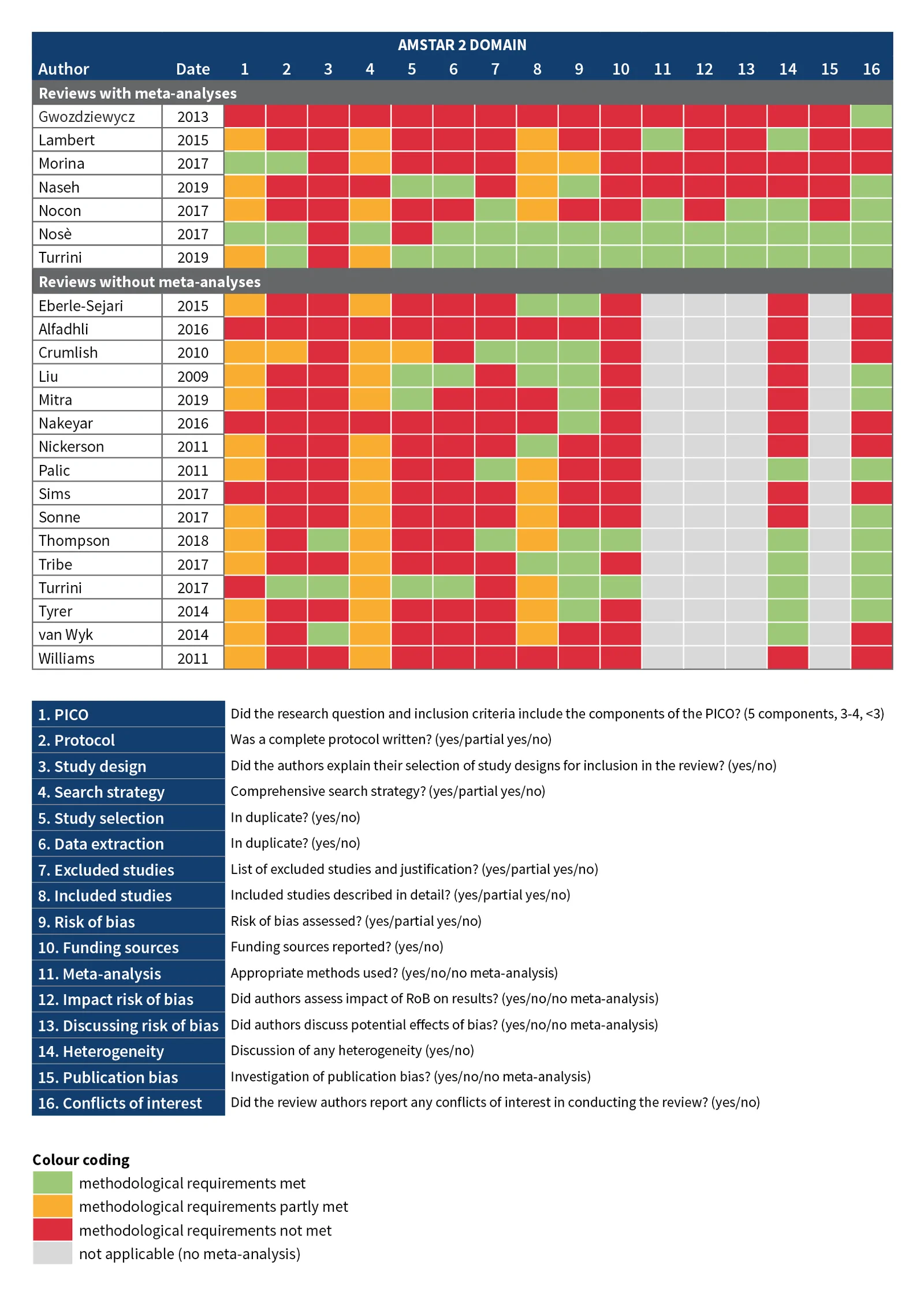
Quality assessment AMSTAR 2.

### Protocol

Four reviews reported a complete protocol containing the main elements and key decisions of a systematic review (Morina 2019; Nosè 2017; Turrini 2017; Turrini 2019), one review had a protocol with some elements missing (Crumlish 2010), and the other 18 reviews had no registered or published protocol.

### Search strategy

One review described a comprehensive search strategy (Nosè 2017), 18 reviews met some of the requirements, and four were missing several key elements (Alfadhli 2016; Gwozdziewycz 2013; Nakeyar 2016; Naseh 2019).

### Study selection

Study screening and selection was performed in duplicate, or partly in duplicate with good agreement between reviewers, for five reviews (Liu 2009a; Mitra 2019; Naseh 2019; Turrini 2017; Turrini 2019). Four reviews did not comprehensively describe all included studies (Alfadhli 2016; Mitra 2019; Nakeyar 2016; Williams 2011) and, for 17 reviews, no list of excluded studies with reasons for exclusion was provided.

### Data extraction

Data extraction was performed in duplicate for five reviews (Liu 2009a; Naseh 2019; Nosè 2017; Turrini 2017; Turrini 2019). In the other 18 reviews, data extraction was not done in duplicate.

### Risk of bias

Risk of bias was not assessed in all reviews. Eleven reviews reported on the key criteria for RCTs (generation of the allocation sequence, selection bias) or non‐RCTs (methods, selection bias). Four reviews also reported on funding sources of included studies (Nosè 2017; Thompson 2018; Turrini 2017; Turrini 2019).

### Meta‐analysis

Fifteen reviews did not perform meta‐analyses, and various domains of AMSTAR 2, therefore, do not apply to these reviews. Three reviews performed meta‐analyses but did not use appropriate methods, for example without a justification for combining studies (Gwozdziewycz 2013; Morina 2019; Naseh 2019).

Of the eight reviews in which meta‐analyses were reported, in three reviews the impact of potential biases on the results was discussed (Nocon 2017; Nosè 2017; Turrini 2019). Two reviews with meta‐analyses assessed the risk of publication bias (Nosè 2017; Turrini 2019).

Out of the 15 reviews for which no meta‐analyses were carried out, ten included a discussion of the potential impacts of heterogeneity of the evidence.

### Conflicts of interest

Thirteen reviews either reported any conflicts of interest or reported that the authors had no conflicts of interest.

### Characteristics of high‐quality systematic reviews

Two systematic reviews with meta‐analyses received the highest quality assessment rating for 14 out of 16 domains (Nosè 2017) and 13 out of 16 domains (Turrini 2019). These reviews were conducted by authors from the same institution, with overlap in the author team. Their inclusion criteria specified either RCTs only (Turrini 2019) or RCTs and non‐randomised trials with a control group (Nosè 2017). Both specified in their eligibility criteria that refugees and asylum seekers were included, either adults only (Nosè 2017) or of all ages (Turrini 2019). Together these reviews covered primary studies of psychological, social, or rehabilitation interventions for PTSD (Nosè 2017) and diagnoses or symptoms of PTSD, depression, and anxiety (Turrini 2019). Any comparators were eligible for inclusion. Out of the 40 references included in these two reviews, there were 28 unique primary studies.

### Effect of interventions

In this overview of systematic reviews, we did not report on the effectiveness of interventions in the included reviews.

## Discussion

### Summary of main results

This overview of systematic reviews included 23 completed reviews and 15 protocols of reviews registered in PROSPERO, which were planned and/or ongoing.

The 23 included systematic reviews comprised 336 references, of which 175 were unique primary studies. All were published between 2009 and 2019. Four systematic reviews included RCTs only. Reviews included studies from any setting, except for one review of studies from high‐income countries and one review of studies from developed countries. As for the population, inclusion criteria were more often specific in their stated inclusion of refugees than asylum seekers or internally displaced persons (Figure).

Most reviews focused on psychological therapies, and there was more evidence on interventions for the treatment of PTSD or trauma‐related symptoms from reviews of any age or adults only than there was for children, people with depression or anxiety, or prevention or mental health promotion (Figure). Pharmacological treatments were considered in only two reviews. Interventions most frequently reported in reviews included CBT‐based approaches, integrative and interpersonal therapies, trauma therapies (including testimony therapy), and creative therapies. Less evidence was available for transdiagnostic therapy, psychodynamic therapy, education, and medication. Review elements compromising the methodological quality of the reviews included the absence of reference to a review protocol, the lack of a comprehensive search strategy, and single screening and/or data extraction of studies included in the review.

Most of the 15 review protocols of ongoing systematic reviews included studies from any setting on interventions for the treatment of common mental disorders. Specific settings included Europe, high‐income countries, low‐ and middle‐income countries and low‐income countries in the Middle East. Study participant eligibility criteria specified refugees, or refugees and asylum seekers, but only four out of 15 protocols listed internally displaced persons as participants eligible for inclusion. Interventions were most often aimed at treating (symptoms of) PTSD or trauma, or the reviews focussed on interventions for mental health in general. Where interventions eligible for inclusion in the reviews were specified, these included various types of psychological therapy, preventative interventions, and community‐based interventions. For the majority of protocols, any comparator was eligible for inclusion.

### Overall completeness and applicability of evidence

A wide range of research questions could be asked in relation to mental health promotion, prevention, and treatment of common mental disorders for refugees, asylum seekers, and internally displaced persons. This overview of systematic reviews shows that some of these questions have received limited attention in reviews published to date. We did not identify many reviews seeking to assess the efficacy of treatments for children or for depression and anxiety (Figure). Inclusion criteria of reviews often did not include internally displaced persons, and few of the included primary studies evaluated pharmacological treatments. The difference between mental health promotion, prevention, and treatment of common mental disorders was usually not explicitly made by review authors, but most reviews included interventions typically delivered as treatment, although some can and are used as preventive interventions.

These gaps in the evidence partly reflect the availability of primary data and partly reflect decisions made in the design of the reviews. For example, the greater availability of data on therapies for the treatment of PTSD rather than anxiety or depression appears to be influenced by a greater evidence base for PTSD in this population. The absence of internally displaced persons from many reviews however is likely to be due to the restricted selection criteria and search terms applied by review authors, which often did not include all three groups of involuntary migrants and instead focussed on refugees.

Most systematic reviews included primary research not limited to RCTs. Although the literature appears to include many non‐randomised and uncontrolled study designs, it is unlikely that robust conclusions on the effectiveness of treatments can be drawn from these studies.

We assessed the quality of the reviews using AMSTAR 2. For many of the AMSTAR 2 domains, reviews can only achieve a high rating if review design and methods are clearly reported. A review scoring lower on this quality assessment may therefore either be of poorer methodological quality, suffer from substandard reporting, or both. Although we did not report on the findings from the reviews, it is clear that the majority of included reviews were lacking both in methodological quality and the transparancy of reporting of the review findings. This would hinder any application of the evidence in practice. For example, without evidence of a comprehensive search strategy, it is unclear whether all relevant primary studies have been included. The lack of reporting on conflicts of interest of review authors makes it difficult to assess the risk of bias affecting review findings.

### Quality of the evidence

Most reviews did not register or publish a protocol. This made it difficult to appraise the methodology of the reviews. For most reviews, participants, interventions, comparators, and outcomes (PICO) were not made explicit, the study design was not fully explained, and the search strategy was not comprehensive (Figure). Most author teams did not perform study selection and screening in duplicate. Excluded studies were not usually reported, and included studies were not always described in sufficient detail. For reviews in which a meta‐analysis was performed, there was evidence that methods applied were not appropriate in three out of eight reviews. In most reviews, potential limitations of primary studies were discussed, but formal 'Risk of bias' assessments, including reporting of primary study funding sources, were not carried out. For ten out of 23 reviews, no conflict of interest statement was included.

Two systematic reviews were ranked as high‐quality across the majority of the assessed domains and may therefore be more informative to future research on this topic (Nosè 2017;Turrini 2019). In line with other reviews included in this overview, these two reviews focussed on refugees and asylum seekers, and included either adults or participants of all ages. One review covered interventions for PTSD only, while the other included diagnoses or symptoms of PTSD, depression, and anxiety.

### Potential biases in the overview process

It is possible that we did not identify all relevant reviews and review protocols. Protocols were only searched through the PROSPERO website, while authors may have registered review protocols on other online platforms. Reviews were only included if the title or abstract specified that a 'systematic review' was conducted and if there was evidence of a systematic search including a search strategy. Reviews on a slightly different population, for example, of studies conducted with people living in settings of humanitarian crises, could be informative for the population of involuntary migrants. We did not include such reviews unless it was made explicit that participants were refugees, asylum seekers, or internally displaced persons.

The list of primary studies from the included reviews is not a comprehensive overview of the literature. Other relevant studies will have been published that were not included in reviews. This overview of systematic reviews will guide the development of new Cochrane reviews, to identify these primary studies.

A limitation of our overview is that we could not obtain a full‐text manuscript for three reviews which were eligible for inclusion in the overview (Anders 2016; Khan 2018; Piegenschke 2019).

Some of the primary studies in the systematic reviews that were included may not be relevant to the topic of this overview. We selected reviews based on the eligibility of the review and review methods, rather than assessing the eligibility of primary studies. For example, some of the studies may discuss mental health of our target population without evaluating an intervention. Also, there may be multiple reports of the same primary study using different manuscript titles among the 175 unique references we identified.

Two of our authors (MP, CB) are also co‐authors for some of the included reviews and review protocols registered in PROSPERO (Nosè 2016; Nosè 2017; Turrini 2017; Turrini 2019; Turrini 2019a). In line with Cochrane guidance, these authors were not involved in any of the data extraction or quality assessments of reviews in this overview. We therefore do not expect the involvement of these authors in the overview to bias our findings.

### Agreements and disagreements with other studies or reviews

In 2017, researchers including two of our co‐authors (MP, CB) published a review of systematic reviews on the prevalence and treatment of common mental disorders in asylum seekers and refugees (Turrini 2017). This umbrella review is included in our overview and comprises 14 systematic reviews on the efficacy of mental health interventions. We identified the same reviews, but excluded six reviews because they were not specifically of involuntary migrant populations. We included eight of the same reviews and 15 additional reviews, including those published after April 2017.

Turrini 2017 reported predominantly on reviews of NET and different types of CBT, and included fewer studies of other interventions such as EMDR, trauma‐focused therapy, testimony therapy, and antidepressants. Primary studies on PTSD were more frequently included in their reviews than studies on depression and anxiety, despite their finding that depression and anxiety were at least as frequent as PTSD among refugees and asylum seekers. These gaps in the evidence are lin line with our findings.

## Authors' conclusions

**Implications for practice**

This overview did not focus on the efficacy of interventions and therefore cannot inform practice directly. It does indicate, however, that the evidence available to decision‐makers in clinical practice and policy is predominantly of limited quality. Only two systematic reviews, reporting on interventions for common mental disorders in refugees and asylum seekers (Turrini 2017) and PTSD for refugees and asylum seekers in high‐income countries (Nosè 2017), achieved a high‐quality rating across most domains.

Most systematic reviews included primary studies other than RCTs, which would make it difficult to draw conclusions on the effectiveness of interventions. For meta‐analyses conducted in reviews with severe methodological limitations, the produced effect estimates may give the impression of evidence readily applicable to practice, while these findings may be biased.

For evidence to inform practice, the evidence base should provide information for populations, settings, and interventions relevant to practice. Our overview shows that important groups such as internally displaced persons, children, and people with depression and anxiety are less likely to be considered in systematic reviews. Evidence on mental health promotion and the prevention of mental health problems, which could be an important avenue for early intervention after resettlement, is largely absent.

**Implications for research**

Many of the 23 included systematic reviews, and the registered review protocols, focused on similar interventions for similar populations. This is illustrated by our finding of 175 unique primary studies among 336 references identified in reviews. Meanwhile, several relevant groups were underrepresented in reviews, leading to gaps in the evidence. Based on the evidence identified in this overview, we see potential for future reviews to address the following research questions for the population of involuntary migrants.

1. What is the efficacy of prevention and treatment of common mental disorders other than PTSD?

Treatment for (symptoms of) PTSD was included in two high‐quality reviews and may therefore not require further evidence synthesis at this time. However, since PTSD is by no means the only mental health problem facing involuntary migrants, reviews of interventions for anxiety and depressive disorders as well as transdiagnostic approaches are required. For established therapies such as CBT and NET, several reviews including a range of primary study designs were identified. To answers questions on the efficacy of interventions, systematic reviews of RCTs would be most useful.

2. What is the efficacy of mental health promotion and prevention and treatment of common mental disorders for children?

Most reviews we identified did not focus on children (Figure), even though different interventions are available and appropriate for children as well as adults and the efficacy of interventions may differ between children and adults.

3. What is the acceptability of interventions for involuntary migrants?

Systematic review authors may wish to consider outcomes such as dropout rates, cultural appropriateness, and cost‐effectiveness. Many of the interventions identified in this overview were not developed for involuntary migrants, which raises unanswered questions about the appropriateness of interventions for this population. Particularly where there are indications of limited efficacy of interventions, measures of acceptability may indicate whether adaptations of interventions to the population or setting are required. Most involuntary migrants live in low‐ and middle‐income countries, where resources are limited and transdiagnostic or task‐shifting approaches may be more appropriate than traditional, resource‐heavy psychological therapies.

To answer any of these research questions, we suggest two ways to strengthen the existing evidence base. Firstly, systematic review authors should consider the explicit inclusion of refugees, asylum seekers, and internally displaced persons in their objectives, selection criteria, and search terms. Internally displaced persons form the largest group of involuntary migrants globally, yet were often not explicitly included in systematic reviews. Secondly, high quality reviews with transparant and complete reporting of review design and methods would aid anyone using these reviews to inform decisions on the implementation of new and existing mental health interventions in practice. For example, the online registration or publication of a review protocol, a description of selection criteria, and an assessment of the quality or risk of bias of included studies are key review elements which should not be missing from any systematic review.

## History

Protocol first published: Issue 10, 2019 Review first published: Issue 9, 2020

## Appendices

### Appendix 1. Searches

4‐Sept‐2019

Search summary

• Ovid MEDLINE (1946 to 3‐Sept‐2019) n = 817

• Ovid Embase (1974 to 2019 Week 35), n = 953

• Ovid PsycINFO (1806 to August Week 4 2019), n = 1190

• Web of Science Core Collection (Science and Social Science Indices) (all years to 4‐Sept‐2019), n = 1406;

• Cochrane Database of Systematic Reviews (CDSR) (www.cochranelibrary.com/) n = 43

• Centre for Reviews and Dissemination (CRD) Databases (archived) (www.crd.york.ac.uk/crdweb/), n = 321

• DoPHER (Database of Promoting Health Effectiveness Reviews) (https://eppi.ioe.ac.uk/webdatabases4/Intro.aspx?ID=9), n = 42

• Health Evidence (www.healthevidence.org/), n = 37

Total = 4809 Duplicates removed = 1314 Records to screen = 3495

Other database searches:

• ProQuest PTSDpubs ((1910 to 4‐Sept‐2019), n = 1210

• NIHR Journals Library – Health Technology Assessment (www.journalslibrary.nihr.ac.uk/HTA/#/) (n = 528)

• 3ie International Initiative for Impact Evaluation: Evidence Hub ‐ Systematic Review Repository (https://www.3ieimpact.org/evidence-hub/systematic-review-repository) n = 286

• Epistemonikos (www.epistemonikos.org/) n = 1026

• PROSPERO (https://www.crd.york.ac.uk/prospero/) n = 530

***************************************************************

SEARCH STRATEGIES

Ovid MEDLINE(R) and Epub Ahead of Print, In‐Process & Other Non‐Indexed Citations and Daily <1946 to September 03, 2019>

Search Strategy:

‐‐‐‐‐‐‐‐‐‐‐‐‐‐‐‐‐‐‐‐‐‐‐‐‐‐‐‐‐‐‐‐‐‐‐‐‐‐‐‐‐‐‐‐‐‐‐‐‐‐‐‐‐‐‐‐‐‐‐‐‐‐‐‐‐‐‐‐‐‐‐‐‐‐‐‐‐‐‐‐

1 refugees/ (9495)

2 "transients and migrants"/ (10956)

3 "emigrants and immigrants"/ or undocumented immigrants/ (11318)

4 human migration/ or "emigration and immigration"/ (25771)

5 *vulnerable populations/ and (psychology or prevention & control or therapy or rehabilitation).fs. (1834)

6 Acculturation/ (6168)

7 asylum.ti,ab,kf. (3267)

8 refugee*.ti,ab,kf. (9895)

9 (migrant? or immigrant? or emigrant?).ti,ab,kf. (40795)

10 (force? adj2 (migrat* or immigrat* or emigrat*)).ti,ab,kf. (567)

11 (displac* adj1 (internal* or forced or mass or person* or people* or population*)).ti,ab,kf. (1871)

12 floating population.ti,ab,kf. (232)

13 exp "Warfare and Armed Conflicts"/ (44997)

14 Torture/ and (psychology or prevention & control or therapy or rehabilitation).fs. (878)

15 exp Disasters/ and (psychology or prevention & control or therapy or rehabilitation).fs. (22737)

16 (humanitarian adj3 (aid or affair* or agenc* or assistance or catastrophe* or crisis or crises or disaster* or effort* or emergenc* or evacuation* or integration or reintegration or mission or organization* or organisation* or program* or relief or setting* or support* or task force or work*)).ti,ab,kf. (2371)

17 (genocide or armed conflict* or mass execution* or mass violence).ti,ab,kf. (1846)

18 (cataclysmic or catastroph* or natural disaster* or drought* or earthquake* or mass evacuation* or famine* or flood or floods or hurricane or cyclone* or landslide* or land slide* or mass casualt* or tsunami* or tidal wave* or volcano*).ti,ab,kf. (70799)

19 (torture* or (politic* adj2 (detention or detainee? or persecut* or prison* or imprison* or violen*))).ti,ab,kf. (2614)

20 ((war or warfare) adj5 (abuse* or crime* or rape* or surviv* or victim*)).ti,ab,kf. (2288)

21 (postconflict* or post conflict*).ti,ab,kf. (908)

22 (Medecin? San? Front* or Red Cross or Red Crescent).mp. (5107)

23 or/1‐22 (217587)

24 Mental Health/ (34753)

25 mental disorders/ (157379)

26 anxiety disorders/ or agoraphobia/ or anxiety, separation/ or neurocirculatory asthenia/ or neurotic disorders/ or panic disorder/ or phobic disorders/ or phobia, social/ (65909)

27 mood disorders/ or depressive disorder/ or depression, postpartum/ or depressive disorder, major/ or depressive disorder, treatment‐resistant/ or dysthymic disorder/ or premenstrual dysphoric disorder/ or seasonal affective disorder/ or cyclothymic disorder/ (117393)

28 "trauma and stressor related disorders"/ or adjustment disorders/ or stress disorders, traumatic/ or combat disorders/ or psychological trauma/ or stress disorders, post‐traumatic/ or stress disorders, traumatic, acute/ (37446)

29 (mental* or psychiatr*).ti,kf. (237219)

30 (mental* adj (health* or ill* or well* or disease* or disorder*)).ti,ab,kf. (199856)

31 (depress* or affective disorder? or affective symptom* or dysthymi* or mood? or anxiety or agoraphobi* or panic or phobi*).ti,ab,kf. (579823)

32 (PTSD or ((posttrauma* or post‐trauma* or post trauma*) adj3 (stress* or disorder? or psych* or symptom*)) or acute* stress* or traumatic* stress* or stress disorder? or combat disorder? or war neuros*).ti,ab,kf. (44360)

33 (psycho* adj (stress* or distress*)).ti,ab,kf. (33366)

34 (emotional adj (adjustment* or disorder*)).ti,ab,kf. (3531)

35 (grief or grieving).mp. (12133)

36 or/24‐35 (997988)

37 (systematic or structured or evidence or trials or studies).ti. and ((review or overview or look or examination or update* or summary).ti. or review.pt.) (188817)

38 (0266‐4623 or 1469‐493X or 1366‐5278 or 1530‐440X or 2046‐4053).is. (17991)

39 meta‐analysis.pt. or (meta‐analys* or meta analys* or metaanalys* or meta synth* or meta‐synth* or metasynth*).ti,ab,kf,hw. (183430)

40 ((systematic or meta) adj2 (analys* or review)).ti,kf. or ((systematic* or quantitativ* or methodologic*) adj5 (review* or overview*)).ti,ab,kf,sh. or (quantitativ$ adj5 synthesis$).ti,ab,kf,hw. (241591)

41 (integrative research review* or research integration).tw. or scoping review?.ti,kf. or (review.ti,kf,pt. and (trials as topic or studies as topic).hw.) or (evidence adj3 review*).ti,ab,kf. (184582)

42 review.pt. and ((medline or medlars or embase or pubmed or scisearch or psychinfo or psycinfo or psychlit or psyclit or cinahl or electronic database* or bibliographic database* or computeri#ed database* or online database* or pooling or pooled or mantel haenszel or peto or dersimonian or der simonian or fixed effect or ((hand adj2 search*) or (manual* adj2 search*))).tw,hw. or (retraction of publication or retracted publication).pt.) (145629)

43 or/37‐42 (518669)

44 23 and 36 and 43 (817)

***************************

Ovid Embase <1974 to 2019 Week 35>

Search Strategy:

‐‐‐‐‐‐‐‐‐‐‐‐‐‐‐‐‐‐‐‐‐‐‐‐‐‐‐‐‐‐‐‐‐‐‐‐‐‐‐‐‐‐‐‐‐‐‐‐‐‐‐‐‐‐‐‐‐‐‐‐‐‐‐‐‐‐‐‐‐‐‐‐‐‐‐‐‐‐‐‐

1 refugee camp/ or refugee/ (11887)

2 asylum seeker/ (779)

3 migrant/ or emigrant/ or immigrant/ (22014)

4 *migration/ or *immigration/ (21261)

5 asylum.ti,ab,kw. (3571)

6 refugee*.ti,ab,kw. (10834)

7 (migrant? or immigrant? or emigrant?).ti,ab,kw. (44883)

8 (force? adj2 (migrat* or immigrat* or emigrat*)).ti,ab,kw. (613)

9 (displac* adj1 (internal* or forced or mass or person* or people* or population*)).ti,ab,kw. (2071)

10 floating population.ti,ab,kw. (220)

11 ((post or after) adj migrat*).ti,ab,kw. (1379)

12 exp warfare/ (17287)

13 torture/ or torture survivor/ (2767)

14 *human rights/ (8635)

15 disaster/ or mass disaster/ or natural disaster/ or relief work/ or rescue work/ (28079)

16 natural disaster/ (2704)

17 (humanitarian adj3 (aid or affair* or agenc* or assistance or catastrophe* or crisis or crises or disaster* or effort* or emergenc* or evacuation* or integration or reintegration or mission or organization* or organisation* or program* or relief or setting* or support* or task force or work*)).ti,ab,kw. (2601)

18 (genocide or armed conflict* or mass execution* or mass violence).ti,ab,kw. (1972)

19 (cataclysmic or catastrophe? or natural disaster* or drought* or earthquake* or mass evacuation* or famine* or flood or floods or hurricane or cyclone* or landslide* or land slide* or mass casualt* or tsunami* or tidal wave* or volcano*).ti,ab,kw. (57296)

20 *"geographic and geological phenomena"/ or avalanche/ or earthquake/ or landslide/ or tsunami/ (11049)

21 *environmental impact/ or desertification/ or drought/ or flooding/ or mass extinction/ or sea level rise/ (20969)

22 (torture* or (politic* adj2 (detention or detainee? or persecut* or prison* or imprison* or violen*))).ti,ab,kw. (3044)

23 ((war or warfare) adj5 (abuse* or crime* or rape* or surviv* or victim*)).ti,ab,kw. (1384)

24 (postconflict* or post conflict*).ti,ab,kw. (971)

25 (Medecin? San? Front* or Red Cross or Red Crescent).mp. (7497)

26 or/1‐25 (198294)

27 mental health/ or community mental health/ or psychological well‐being/ (144672)

28 mental disease/ (209514)

29 exp anxiety disorder/ (226627)

30 exp mood disorder/ (488847)

31 (mental* or psychiatr*).ti,kw. (251648)

32 (mental* adj (health* or ill* or well* or disease* or disorder*)).ti,ab,kw. (240632)

33 (depress* or affective disorder? or affective symptom* or dysthymi* or mood? or anxiety or agoraphobi* or panic or phobi*).ti,ab,kw. (780733)

34 (PTSD or ((posttrauma* or post‐trauma* or post trauma*) adj3 (stress* or disorder? or psych* or symptom*)) or acute* stress* or traumatic* stress* or stress disorder? or combat disorder? or war neuros*).ti,ab,kw. (57598)

35 (psycho* adj (stress* or distress*)).ti,ab,kw. (45367)

36 (emotional adj (adjustment* or disorder*)).ti,ab,kw. (4731)

37 (grief or grieving).mp. (13850)

38 (counsel* or psychotherap*).ti,hw,kw. (244512)

39 psychiatry.ec. (1005443)

40 or/27‐39 (2038174)

41 systematic review/ or meta analysis/ or network meta‐analysis/ (306369)

42 ((systematic or structured or evidence or trials or studies) and (review or overview or look or examination or update* or summary)).ti. (157903)

43 (0266‐4623 or 1469‐493X or 1366‐5278 or 1530‐440X or 2046‐4053).is. (26008)

44 (systematic review? or evidence report* or technology assessment?).jw. (28506)

45 (meta‐analys* or meta analys* or metaanalys* or meta synth* or meta‐synth* or metasynth*).ti,ab,kw,hw. (273054)

46 ((systematic or meta) adj2 (analys* or review)).ti,kw. or ((systematic* or quantitativ* or methodologic*) adj5 (review* or overview*)).ti,ab,kw,sh. or (quantitativ* adj5 synthes*).ti,ab,kw,hw. (343535)

47 exp "clinical trial (topic)"/ and review.ti,kw,pt. (112587)

48 (integrative research review* or research integration).ti,ab,kw. or scoping review?.ti,kw. or (evidence adj3 review*).ti,ab,kw. (57952)

49 review.pt. and (medline or medlars or embase or pubmed or scisearch or psychinfo or psycinfo or psychlit or psyclit or cinahl or electronic database* or bibliographic database* or computeri#ed database* or online database* or pooling or pooled or mantel haenszel or peto or dersimonian or der simonian or fixed effect or ((hand adj2 search*) or (manual* adj2 search*))).ti,ab,kw,hw. (131711)

50 review.pt. and ((evidence based adj (medicine or practice)) or (outcome? adj (assessment or research)) or treatment outcome).hw. (202907)

51 or/41‐50 (752956)

52 26 and 40 and 51 (953)

***************************

Ovid PsycINFO <1806 to August Week 4 2019>

Search Strategy:

‐‐‐‐‐‐‐‐‐‐‐‐‐‐‐‐‐‐‐‐‐‐‐‐‐‐‐‐‐‐‐‐‐‐‐‐‐‐‐‐‐‐‐‐‐‐‐‐‐‐‐‐‐‐‐‐‐‐‐‐‐‐‐‐‐‐‐‐‐‐‐‐‐‐‐‐‐‐‐‐

1 refugees/ (5546)

2 asylum seeking/ or political asylum/ (501)

3 human migration/ or *immigration/ (23225)

4 acculturation/ (9545)

5 asylum.ti,ab,id. (3028)

6 refugee*.ti,ab,id. (7871)

7 (migrant? or immigrant? or emigrant?).ti,ab,id. (33302)

8 (force? adj2 (migrat* or immigrat* or emigrat*)).ti,ab,id. (367)

9 (displac* adj1 (internal* or forced or mass or person* or people* or population*)).ti,ab,id. (920)

10 floating population.ti,ab,id. (20)

11 Belonging/ (2131)

12 ((post or after) adj migrat*).ti,ab,id. (564)

13 *war/ (9072)

14 torture/ or persecution/ (1682)

15 disasters/ or natural disasters/ (8702)

16 Human Rights/ (6052)

17 Freedom/ (2960)

18 (humanitarian adj3 (aid or affair* or agenc* or assistance or catastrophe* or crisis or crises or disaster* or effort* or emergenc* or evacuation* or integration or reintegration or mission or organization* or organisation* or program* or relief or setting* or support* or task force or work*)).ti,ab,id. (1141)

19 (genocide or armed conflict* or mass execution* or mass violence).ti,ab,id. (2898)

20 (cataclysmic or catastrophe? or natural disaster* or drought* or earthquake* or mass evacuation* or famine* or flood or floods or hurricane or cyclone* or landslide* or land slide* or mass casualt* or tsunami* or tidal wave* or volcano*).ti,ab,id. (10928)

21 (torture* or (politic* adj2 (detention or detainee? or persecut* or prison* or imprison* or violen*))).ti,ab,id. (4348)

22 ((war or warfare) adj5 (abuse* or crime* or rape* or surviv* or victim*)).ti,ab,id. (1616)

23 (postconflict* or post conflict*).ti,ab,id. (1241)

24 (Medecin? San? Front* or Red Cross or Red Crescent).mp. (541)

25 or/1‐24 (90692)

26 (meta analysis or "systematic review").md. (39590)

27 meta analysis/ (4493)

28 ((systematic or structured or evidence or trials or studies) and (review or overview or look or examination or update* or summary)).ti. (25653)

29 (meta‐analys* or meta analys* or metaanalys* or meta synth* or meta‐synth* or metasynth*).ti,ab,id,hw. (33131)

30 ((systematic or meta) adj2 (analys* or review)).ti,id. or ((systematic* or quantitativ* or methodologic*) adj5 (review* or overview*)).ti,ab,id,sh. or (quantitativ* adj5 synthes*).ti,ab,id,hw. (50353)

31 (integrative research review* or research integration).ti,ab,id. or scoping review?.ti,id. or (evidence adj3 review*).ti,ab,id. (17390)

32 "literature review"/ and (medline or medlars or embase or pubmed or scisearch or psychinfo or psycinfo or psychlit or psyclit or cinahl or electronic database* or bibliographic database* or computeri#ed database* or online database* or pooling or pooled or mantel haenszel or peto or dersimonian or der simonian or fixed effect or ((hand adj2 search*) or (manual* adj2 search*))).ti,ab,id,hw. (22316)

33 ((systematic or structured or evidence or trials or studies) adj3 review*).ti,ab,id. and (evidence based practice or treatment outcomes or mental health program evaluation).sh. (3195)

34 or/26‐33 (104031)

35 25 and 34 (1190)

***************************

Web of Science Core Collection (All years to 4‐Sept‐2019)

TOPIC: ((asylum or refugee* or migrant* or immigrant* or emigrant* or acculturation or "displac* person*" or "displac* people*" or "displac* population*" or "internal displacement*" or "floating population" or humanitarian or "human rights" or genocide or "armed conflict*" or "mass execution*" or "mass violence" or cataclysmic or catastrophe* or "natural disaster*" or drought* or earthquake* or "mass evacuation*" or famine* or flood or floods or hurricane* or cyclone* or landslide* or "land slide*" or "mass casualt*" or tsunami* or "tidal wave*" or volcano* or torture* or "politic* detention*" or "politic* detainee*" or "politic* persecut*" or "politic* prison*" or "politic* imprison*" or "politic* violen*" or ((war or warfare) AND (abuse* or crime* or rape* or surviv* or victim*)) or postconflict* or "post conflict*" or (Medecin* San* Front*) or "Red Cross" or "Red Crescent")) AND TOPIC: ((psychiatr* or "mental* health*" or "mental* ill*" or "mental* well*" or "mental* disease*" or "mental* disorder*" or depress* or "affective disorder*" or "affective symptom*" or dysthymi* or mood or moods or anxiety or agoraphobi* or panic or phobi* or PTSD or "posttrauma* stress*" or "post‐trauma* stress*" or "post trauma* stress" or "psych* trauma*" or "psych* stress*" or "psych* distress*" or "acute* stress*" or "traumatic* stress*" or "stress disorder*" or "combat disorder*" or "war neuros*" or "emotional adjustment*" or "emotional disorder*" or grief or grieving)) AND TOPIC: ((meta‐analys* or "meta analys*" or metaanalys* or "meta synth*" or meta‐synth* or metasynth* or “systematic* review*” or “systematic* overview*” or “systematic* analys*” or “systematic* synthes*” or "integrative research review*" or "research integration" or "scoping review*" or (evidence AND review*) or “quantitativ* analys*” or “quantitativ* review*” or “quantitativ* synthes*”))

Indexes = SCI‐EXPANDED, SSCI Timespan = All years n = 1406

***************************

Cochrane Database of Systematic Reviews (CDSR), Issue 9, 2019

Title/Abstract/Keywords: ((asylum or refugee* or migrant* or immigrant* or emigrant* or acculturation or "displac* person*" or "displac* people*" or "displac* population*" or "internal displacement*" or "floating population" or humanitarian or "human rights" or genocide or "armed conflict*" or "mass execution*" or "mass violence" or cataclysmic or catastrophe* or "natural disaster*" or drought* or earthquake* or "mass evacuation*" or famine* or flood or floods or hurricane* or cyclone* or landslide* or "land slide*" or "mass casualt*" or tsunami* or "tidal wave*" or volcano* or torture* or "politic* detention*" or "politic* detainee*" or "politic* persecut*" or "politic* prison*" or "politic* imprison*" or "politic* violen*" or ((war or warfare) AND (abuse* or crime* or rape* or surviv* or victim*)) or postconflict* or "post conflict*" or (Medecin* San* Front*) or "Red Cross" or "Red Crescent”)) n=43

[Reviews (38), Protocols (3), Clinical Answers (2)]

***************************

Centre for Reviews and Dissemination (CRD) Databases (archived) (www.crd.york.ac.uk/crdweb/)

1. MeSH DESCRIPTOR Refugees EXPLODE ALL TREES (11)

2. MeSH DESCRIPTOR Transients and Migrants EXPLODE ALL TREES (11)

3. MeSH DESCRIPTOR Emigrants and Immigrants EXPLODE ALL TREES (25)

4. MeSH DESCRIPTOR Undocumented Immigrants EXPLODE ALL TREES (0)

5. MeSH DESCRIPTOR Human Migration EXPLODE ALL TREES (22)

6. MeSH DESCRIPTOR Acculturation EXPLODE ALL TREES (3)

7. (asylum) (6)

8. (refugee*) (18)

9. (migrant* ) OR (immigrant*) OR (emigrant*) (81)

10. ((force* NEAR (migrat* or immigrat* or emigrat*))) (0)

11. ((displac* NEAR (internal* or forced or mass or person* or people* or population*))) (9)

12. (floating population) (2)

13. MeSH DESCRIPTOR Warfare and Armed Conflicts EXPLODE ALL TREES (21)

14. MeSH DESCRIPTOR Torture EXPLODE ALL TREES (0)

15. MeSH DESCRIPTOR Disasters EXPLODE ALL TREES (104)

16. (humanitarian) (8)

17. (genocide or armed conflict* or mass execution* or mass violence) (1)

18. (cataclysmic or catastroph* or natural disaster* or drought* or earthquake* or mass evacuation* or famine* or flood or floods or hurricane or cyclone* or landslide* or land slide* or mass casualt* or tsunami* or tidal wave* or volcano*) (57)

19. ((torture* or (politic* NEAR (detention or detainee? or persecut* or prison* or imprison* or violen*)))) (2)

20. (war or warfare) (24)

21. (postconflict* or post conflict*) (1)

22. (Medecin* San* Front* or Red Cross or Red Crescent) (33)

23. #1 OR #2 OR #3 OR #4 OR #5 OR #6 OR #7 OR #8 OR #9 OR #10 OR #11 OR #12 OR #13 OR #14 OR #15 OR #16 OR #17 OR #18 OR #19 OR #20 OR #21 OR #22 (321)

***************************

DoPHER (Database of Promoting Health Effectiveness Reviews)

(https://eppi.ioe.ac.uk/webdatabases4/Intro.aspx?ID=9) (4‐Sept‐2019)

1. refugee (8)

2. refugees (4)

3. asylum (3)

4. displaced persons (1)

5. displaced people (1)

6. displaced population (0)

7. displaced populations (0)

8. migrants (12)

9. migrant population (2)

10. immigrants (9)

11. emigrants (0)

12. humanitarian (11)

13. acculturation (4)

14. or/1‐13, n=42

***************************

Health Evidence (www.healthevidence.org/) (4‐Sept‐2019)

refugee OR refugees OR asylum OR “displaced persons” OR “displaced people” OR “displaced population” OR “displaced populations” OR migrants OR “migrant population” OR acculturation OR humanitarian n = 37

***************************************************************

OTHER DATABASE SEARCHES

Proquest PTSDPubs (1910 to 4‐Sept‐2019)

S1 MAINSUBJECT.EXACT.EXPLODE(“Migrants") OR MAINSUBJECT.EXACT.EXPLODE("Acculturation") OR noft((asylum or refugee* or migrant* or immigrant* or emigrant* or migration OR immigration OR emigration)) OR noft((displac* and (internal* or forced or mass or person* or people* or population* or “floating population”)))

S2 MAINSUBJECT.EXACT.EXPLODE("War") OR MAINSUBJECT.EXACT(“Torture") OR (MAINSUBJECT.EXACT.EXPLODE("Natural Disasters") OR MAINSUBJECT.EXACT(“Disasters")) OR noft(humanitarian or “human rights”) OR noft((genocide or (armed W/2 conflict*) or (mass W/2 execution*) or "mass violence”)) OR noft(((cataclysmic or catastrophe? or (natural W/2 disaster*) or drought* or earthquake* or (mass W/2 evacuation*) or famine* or flood or floods or hurricane or cyclone* or landslide* or (land W/2 slide*) or (mass W/2 casualt*) or tsunami* or (tidal W/2 wave*) or volcano*))) OR noft((torture* or (politic* and (detention or detainee? or persecut* or prison* or imprison* or violen*)))) OR noft(((war or warfare) and (abuse* or crime* or rape* or surviv* or victim*))) OR noft( (postconflict* or (post w/2 conflict*))) OR ((Medecin* San* Front*) or "Red Cross" or "Red Crescent”)

S3 (MAINSUBJECT.EXACT("Literature Review") OR MAINSUBJECT.EXACT("Systematic Review") OR MAINSUBJECT.EXACT("Meta Analysis")) OR noft((meta‐analys* OR (meta w/2 analys*) OR metaanalys* OR (meta w/2 synth*) OR meta‐synth* OR metasynth*)) OR noft(((systematic* OR quantitativ* OR methodologic*) AND (analys* OR synthes* OR review* OR overview*))) OR noft(("integrative research review*" OR "research integration" OR "scoping review*" OR (evidence W/3 review*)))

S4 ((S1OR S2) AND S3), n = 1210

***************************

NIHR Journals Library – Health Technology Assessment (www.journalslibrary.nihr.ac.uk/HTA/#/)

#1 refugee OR refugees OR asylum (4)

#2 “displaced persons” OR “displaced people” OR “displaced populations” (0)

#3 migrant OR migrants (6)

#5 (acculturation (0)

#4 humanitarian (2)

Records screened in‐situ

***************************

3ie International Initiative for Impact Evaluation: Evidence Hub ‐ Systematic Review Repository

https://www.3ieimpact.org/evidence-hub/systematic-review-repository

Search: refugee OR refugees OR asylum OR “displaced persons” OR “displaced people” OR “displaced populations” OR migrants OR “migrant population” OR acculturation OR humanitarian n = 181

***************************

Epistemonikos

#1 refugee OR refugees OR asylum (271)

#2 “displaced persons” OR “displaced people” OR “displaced populations” (35)

#3 migrant OR migrants OR acculturation (490)

#4 humanitarian (61)

Records screened in‐situ

***************************

Prospero

#1 refugee OR refugees OR asylum (310)

#2 displaced persons OR displaced people OR displaced populations (58)

#3 migrant OR migrants OR acculturation OR humanitarian (389)

#4 (#1 OR #2 OR #3) (530)

Records screened in‐situ

***************************************************************

### Appendix 2. Primary studies of included reviews

**Table:**

**Reference for primary study**
Acarturk C, Konuk E, Cetinkaya M, Senay I, Sijbrandij M, Cuijpers P, et al. EMDR for Syrian refugees with posttraumatic stress disorder symptoms: results of a pilot randomized controlled trial. European Journal of Psychotraumatology 2015;6:1‐9.
Acarturk C, Konuk E, Cetinkaya M, Senay I, Sijbrandij M, Gulen B, et al. The efficacy of eye movement desensitization and reprocessing for post‐traumatic stress disorder and depression among Syrian refugees: results of a randomized controlled trial. Psychological Medicine 2006;46:2583‐593.
Adenauer H, Catani C, Gola H, Keil J, Ruf M, Schauer M, et al. Narrative exposure therapy for PTSD increases top‐down processing of aversive stimuli—evidence from a randomized controlled treatment trial. BMC Neuroscience 2011;12:127.
Adhikari P. The plight of the forgotten ones: civil war and forced migration. International Studies Quarterly 2012;56:590‐606.
Admirand P. The ethics of displacement and migration in the Abrahamic faiths: enlightening believers and aiding public policy. Journal of Ethnic and Migration Studies 2014;40:671‐87.
Ager A, Akesson B, Stark L, Flouri E, Okot B. The impact of the schoolbased psychosocial structured activities (PSSA) program on conflict‐affected children in Northern Uganda. Journal of Child Psychology and Psychiatry 2011;52:1124–33.
Ager A. Tensions in the psychosocial discourse: implications for the planning of interventions with war‐affected populations. Development in Practice 1997;7(4):402–7.
Agier M. Between war and city. Towards an urban anthropology of refugee camps. Ethnography 2002;3:317‐41.
Akhtar P. Project report: therapeutic effects of music on torture survivors and refugees. Torture 1994;4:121‐23.
Allden K, Jones L, Weissbecker I, Wessells M, Bolton P, Betancourt TS, et al. A. Mental health and psychosocial support in crisis and conflict: report of the mental health working group. Prehospital and Disaster Medicine 2009;24:s217‐27.
Almakhamreh S, Hundt GL. An examination of social work interventions for use with displaced Iraqi households in Jordan. European Journal of Social Work 2012;15:377‐91.
Arcel LT, Popovic S, Kucukalic A, Bravo‐Mehmedbasic A, Ljubotina D, Pusina J, et al. The impact of short‐term treatment on torture survivors: the change in PTSD, other psychological symptoms and coping mechanisms after treatment. In: Arcel, LT, Popovic S, Kacukalic A, Bravo‐Mehmedbasic A. (Eds.). Treatment of torture and trauma survivors in a post‐war society. Association of Rehabilitation and Torture Victims 2003; Centre for Torture Victims (CTV), Sarajevo:135‐57.
Arntz A, Sofi D, Van Breukelen G. Imagery rescripting as treatment for complicated PTSD in refugees: a multiple baseline case series study. Behaviour Research & Therapy 2013;51:274–83.
Atlas M. Experiencing displacement: using art therapy to address xenophobia in South Africa. Development 2009;52:531‐6.
Bader F, Sinha R, Leigh J, Goyal N, Andrews A, Valeeva N, et al. Psychosocial health in displaced Iraqi care‐seekers in non‐governmental organization clinics in Amman, Jordan: An unmet need. Prehospital and Disaster Medicine 2009;24:312‐20.
Badri A, den Borne HW, Crutzen R. Experiences and psychosocial adjustment of Darfuri female students affected by war: An exploratory study. International Journal of Psychology 2013;48:944‐53.
Baker F, Jones C. The effect of music therapy services on classroom behaviours of newly arrived refugee students in Australia – a pilot study. Emotional and Behavioural Difficulties 2006;11:249‐60.
Barrett PM, Moore AF, Sonderegger R. The FRIENDS program for young former‐Yugoslavian refugees in Australia: A pilot study. Behaviour Change 2000;17(3):124‐33.
Barrett PM, Sonderegger R, Xenos S. Using FRIENDS to combat anxiety and adjustment problems among young migrants to Australia: A national trial. Clinical Child Psychology and Psychiatry 2003;8:241–60.
Başoğlu M, Ekblad S, Bäärnhielm S, Livanou M. Cognitive‐behavioral treatment of tortured asylum seekers: a case study. Journal of Anxiety Disorders 2004;18(3):357‐69.
Beehler S, Birman D, Campbell R. The effectiveness of cultural adjustment and trauma services (CATS): Generating practice‐based evidence on a comprehensive, school‐based mental health intervention for immigrant youth. American Journal of Community Psychology 2012;50(1‐2):155–68.
Betancourt TS, Newnham EA, Brennan RT, Verdeli H, Borisova I, Neugebauer R, et al. Moderators of treatment effectiveness for war‐affected youth with depression in northern Uganda. Journal of Adolescent Health 2012;51(6):544–50.
Betancourt TS. Stressors, supports and the social ecology of displacement: Psychosocial dimensions of an emergency education program for Chechen adolescents displaced in Ingushetia, Russia. Culture Medicine and Psychiatry 2005;29(3):309‐40.
Bichescu D, Neuner F, Schauer M, Elbert T. Narrative exposure therapy for political imprisonment‐related chronic posttraumatic stress disorder and depression. Behaviour Research and Therapy 2007;45:2212−220.
Birck A. Torture victims after psychotherapy ‐ a two year follow‐up. Torture 2001;11:55‐58.
Birman D, Beehler S, Harris EM, Everson ML, Batia K, Liataud J, et al. International family, adult, and child enhancement services (FACES): A community‐based comprehensive services model for refugee children in resettlement. American Journal of Orthopsychiatry 2008;78:121‐32.
Bodeker G, Neumann C. Revitalization and development of Karen traditional medicine for sustainable refugee health services at the Thai‐Burma border. Journal of Immigrant & Refugee Studies 2012;10:6‐30.
Boehnlein JK, Kinzie JD, Ben R, Fleck, J. One‐year follow‐up study of posttraumatic stress disorder among survivors of Cambodian concentration camps. American Journal of Psychiatry 1985;142(8):956‐59.
Boehnlein JK, Kinzie JD, Sekiya U, Riley C, Pou K, Rosborough B. A ten‐year treatment outcome study of traumatized Cambodian refugees. Journal of Nervous and Mental Disease 2004;192(10):658–63.
Boehnlein JK, Kinzie JD. Pharmacologic reduction of CNS noradrenergic activity in PTSD: The case for clonidine and prazosin. Journal of Psychiatric Practice 2007;13(2):72‐8.
Bogac C. Place attachment in a foreign settlement. Journal of Environmental Psychology 2009;29:267‐78.
Bolton P, Bass J, Betancourt T, Speelman L, Onyango G, Clougherty K, et al. Interventions for depression symptoms among adolescent survivors of war and displacement in Northern Uganda: a randomized controlled trial. JAMA 2007;298(5):519‐27.
Bolton P, Bass J, Neugebauer R, Verdeli H, Clougherty K, Wickramaratne P, et al. Group interpersonal psychotherapy for depression in rural Uganda: a randomized controlled trial. JAMA 2003; 289(23), 3117–124.
Bolton P, Lee C, Haroz EE, Murray L, Dorsey S, Robinson C, et al. A transdiagnostic community‐based mental health treatment for comorbid disorders: development and outcomes of a randomized controlled trial among Burmese refugees in Thailand. PLoS Medicine 2014;11:1‐16.
Boynton L, Bentley J, Strachan E, Barbato A, Raskind M. Preliminary findings concerning the use of prazosin for the treatment of posttraumatic nightmares in a refugee population. Journal of Psychiatric Practice 2009;15(6):454‐59.
Bradley M. Rethinking refugeehood: statelessness, repatriation, and refugee agency. Review of International Studies 2014;40:101‐23.
Breslau J. Cultures of trauma: anthropological views of posttraumatic stress disorder in international health. Culture, Medicine and Psychiatry 2004;28(2):113‐26.
Briant N, Kennedy A. An investigation of the perceived needs and priorities held by African refugees in an urban setting in a first country of asylum. Journal of Refugee Studies 2004;17(4):437‐59.
Brune M, Haasen C, Krausz M, Yagdiran O, Bustos E, Eisenmann D. Belief systems as coping factors for traumatized refugees: a pilot study. European Psychiatry 2002;17:451‐58.
Buhmann CB, Nordentoft M, Ekstroem M, Carlsson J, Mortensen EL. The effect of flexible cognitive behavioural therapy and medical treatment, including antidepressants on post‐traumatic stress disorder and depression in traumatised refugees: pragmatic randomised controlled clinical trial. British Journal of Psychiatry 2016;208:252‐59.
Bulley D. Inside the tent: Community and government in refugee camps. Security Dialogue 2014;45:63‐80.
Buyer M. Negotiating identity and displacement among the Somali refugees of Cape Town. South African Historical Journal 2008;60(2):226‐41.
Carlsson JM, Mortensen EL, Kastrup M. A follow‐up study of mental health and health‐related quality of life in tortured refugees in multi‐discipilinary treatment. Journal of Nervous and Mental Disease 2005;193:651‐57.
Carlsson JM, Olsen DR, Kastrup M, Mortensen EL. Late mental health changes in tortured refugees in multidisciplinary treatment. Journal of Nervous and Mental Disease 2010;198(11):824‐28.
Catani C, Kohiladevy M, Ruf M, Schauer E, Elbert T, Neuner F. Treating children traumatized by war and tsunami: a comparison between exposure therapy and meditation‐relaxation in North‐East Sri Lanka. BMC Psychiatry 2009;9(22).
Cavic T, Pejovic M. The evaluation of group cognitive psychotherapy for posttraumatic stress disorder. [Evaluacija grupne kognitivne psihoterapije posttraumatskog stresnog poremecaja]. Psihijatrija Danas 2005;37:307–13.
Chatty D, Mansour N, Yassin N. Statelessness and tribal identity on Lebanon’s eastern borders. Mediterranean Politics 2013;18:411‐26.
Cheung P. Somatisation as a presentation in depression and post‐traumatic stress disorder among Cambodian refugees. Australian and New Zealand Journal of Psychiatry 1993;27(3): 422–28.
Curley, B. Siting Sudaneseness: Territory, practice, and identity in Aragi. Refuge 2009;26:183‐90.
d'Ardenne P, Ruaro L, Cestari L, Fakhoury W, Priebe, S. Does interpreter‐mediated CBT with traumatized refugee people work? A comparison of patient outcomes in East London. Behavioural and Cognitive Psychotherap. 2007;35:293‐301.
Davenport CA, Moore WH, Poe SC. Sometimes you just have to leave: domestic threats and forced migration, 1964‐1989. International Interactions 2003;29:27‐55.
DeMartino R, Mollica RF, Wilk V. Monoamine oxidase inhibitors in posttraumatic stress disorder. Promise and problems in Indochinese survivors of trauma. Journal of Nervous and Mental Disease 1995;183(8): 510–15.
Drožđek B, Bolwerk N. Evaluation of group therapy with traumatized asylum seekers and refugees – the Den Bosch model. Traumatology 2010;16:117‐27.
Drožđek B, Kamperman AM, Bolwerk N, Tol WA, Kleber RJ. Group therapy with male asylum seekers and refugees with posttraumatic stress disorder: A controlled comparison cohort study of three day‐treatment programs. Journal of Nervous and Mental Disease 2012;200:758–65.
Drožđek B, Kamperman AM, Tol WA, Knipscheer JW, Kleber RJ. Seven‐year follow‐up study of symptoms in asylum seekers and refugees with PTSD treated with trauma‐focused groups. Journal of Clinical Psychology 2014;70:376–87.
Drožđek B. Follow‐up study of concentration camp survivors from Bosnia‐Herzegovina: Three years later. Journal of Nervous and Mental Disease 2007;185:690‐94.
Dudley S. Feeling at home: Producing and consuming things in Karenni refugee camps on theThai‐Burma border. Population, Space and Place 2011;17:742‐55.
Durà‐Vilà G, Klasen H, Makatini Z, Rahimi Z, Hodes M. Mental health problems of young refugees: duration of settlement, risk factors and community‐based interventions. Clinical Child Psychology and Psychiatry 2013;18:604‐23.
Dybdahl R. Children and mothers in war: an outcome study of a psychosocial intervention program. Child Development 2001;72(4):1214‐30.
Ehntholt KA, Smith PA, Yule W. School‐based cognitive‐behavioural therapy group intervention for refugee children who have experienced war‐related trauma. Clinical Child Psychology and Psychiatry 2005;10(2):235‐50.
Eisenbruch M, de Jong JTVM, van de Put W. Bringing order out of chaos: A culturally competent approach to managing the problems of refugees and victims of organized violence. Journal of Traumatic Stress 2004;17:123‐131.
Ellis BH, Miller AB, Abdi S, Barrett C, Blood E, Betancourt TS. Multi‐tier mental health program for refugee youth. Journal of Consulting and Clinical Psychology 2013;81(1):129‐40.
El‐Shaarawi N. Living an uncertain future: An ethnography of displacement, health, psychosocial well‐being and the search for durable solutions among Iraqi refugees in Egypt [PhD thesis]. Cleveland (US): Case Western Reserve University, 2012.
Ertl V, Pfeiffer A, Schauer E, Elbert T, Neuner F. Community‐implemented trauma therapy for former child soldiers in Northern Uganda: a randomized controlled trial. JAMA 2011;306:503‐12.
Fazel M, Doll H, Stein A. A school‐based mental health intervention for refugee children: An exploratory study. Clinical Child Psychology and Psychiatry 2009;14(2):297‐309.
Felke TP. It’s been twenty years: the case of ethnic Armenian refugees from Nagorno‐Karabakh and Azerbaijan [PhD thesis]. Mansfield (US): University of Connecticut, 2010.
Ferris EG. Abuse of power: sexual exploitation of refugee women and girls. Signs 2007;32: 584‐91.
Fiddian‐Qasmiyeh E. Faith‐based humanitarianism in contexts of forced displacement. Journal of Refugee Studies 2011;24:429‐39.
Folkes CE. Thought field therapy and trauma recovery. International Journal of Emergency Mental Health 2002;4:99‐103.
Fox PG, Rossetti J, Burns KR, Popovich J. Southeast Asian refugee children: a school‐based mental health intervention. International Journal of Psychiatric Nursing Research 2005;11: 1227‐236.
Frances A, Kroll J. Ongoing treatment of a Hmong widow who suffers from pain and depression. Hospital & Community Psychiatry 1989;40(7):691–93.
Franke MFN. Refugee registration as foreclosure of the freedom to move: the virtualisation of refugees’ rights within maps of international protection. Society and Space 2009;27:352‐69.
Geltman PL, Grant‐Knight W, Ellis H, Landgraf JM. The ‘’lost boys” of Sudan: use of health services and functional health outcomes of unaccompanied refugees minors resettled in the US. Journal of Immigrant and Minority Health 2008;10(5):389‐96.
Gregory JL, Prana H. Posttraumatic growth in Cote d’Ivoire refugees using the companion recovery model. Traumatology 2013;19(3):223–32.
Gupta L, Zimmer C. Psychosocial intervention for war‐affected children in Sierra Leone. The British Journal of Psychiatry: The Journal of Mental Science 2008;192(3):212‐16.
Halvorsen JO, Stenmark H. Narrative exposure therapy for posttraumatic stress disorder in tortured refugees: A preliminary uncontrolled trial. Scandinavian Journal of Psychology 2010;51(6):495‐502.
Harding S, Libal K. Iraqi refugees and the humanitarian costs of the Iraq war: what role for social work? International Journal of Social Welfare 2012;21:94‐104.
Harris K, Maxwell C. A needs assessment in a refugee mental health project in North‐East London: extending the counselling model to community support. Medicine, Conflict and Survival 2000;16(2):201‐15.
Hensel‐Dittmann, D, Schauer M, Ruf M, Catani C, Odenwald, M, Elbert T. Treatment of traumatized victims of war and torture: A randomized controlled comparison of narrative exposure therapy and stress inoculation training. Psychotherapy and Psychosomatics 2011;80:345‐52.
Hijazi AM, Lumley MA, Ziadni MS, Haddad L, Rapport LJ, Arnetz BB. Brief narrative exposure therapy for posttraumatic stress in Iraqi refugees: a preliminary randomized clinical trial. Journal of Traumatic Stress 2014;27:314‐22.
Hijazi AM. Narrative exposure therapy to treat traumatic stress in Middle Eastern refugees: a clinical trial [PhD thesis]. Detroit (US): Wayne State University, 2012
Hinton DE, Chhean D, Pich V, Safren SA, Hofmann SG, Pollack MH. A randomized controlled trial of cognitive‐behavior therapy for Cambodian refugees with treatment‐resistant PTSD and panic attacks: a cross‐over design. Journal of Traumatic Stress 2005;18:617‐29.
Hinton DE, Hofmann SG, Pollack MH, Otto MW. Mechanisms of efficacy of CBT for Cambodian refugees with PTSD: improvement in emotion regulation and orthostatic blood pressure response. CNS Neuroscience & Therapeutics 2009;15:255‐63.
Hinton DE, Kredlow MA, Bui E, Pollack MH, Hofmann SG. Treatment change of somatic symptoms and cultural syndromes among Cambodian refugees with PTSD. Depression and Anxiety 2012;29(2):147‐54.
Hinton DE, Pham T, Tran M, Safren SA, Otto MW, Pollack MH. CBT for Vietnamese refugees with treatment‐resistant PTSD and panic attacks: a pilot study. Journal of Traumatic Stress 2004;17:429‐33.
Holmqvist R, Andersen K, Anju T, Alinder B. Change in self‐image and PTSD symptoms in short‐term therapies with traumatized refugees. Psychoanalytic Psychotherapy 2006;20:251−65.
Hunt N, Gakenyi M. Comparing refugees and nonrefugees: the Bosnian experience. Anxiety Disorders 2005;19:717‐23.
Išpanović‐Radojković V, Petrović V, Davis H, Tenjović L, Minčić T. Evaluation of psychosocial intervention with traumatised adolescents. In: Powell S,Durakovic‐Belko E, editors. The psychosocial consequences of war: results of empirical research from the territory of former Yugoslavia. Sarajevo (Bosnia Herzegovina):UNICEF, 2002;268‐71.
Jordans MJD, Semrau M, Thornicroft G, van Ommeren M. Role of current perceived needs in explaining the association between past trauma exposure and distress in humanitarian settings in Jordan and Nepal. British Journal of Psychiatry 2012;201:276‐81.
Kaiser T. Songs, discos and dancing in Kiryandongo, Uganda. Journal of Ethnic and Migration Studies 2006;32:183‐202.
Kalantari M, Yule W, Dyregrov A, Neshatdoost H, Ahmadi SJ. Efficacy of writing for recovery on traumatic grief symptoms of Afghani refugee bereaved adolescents: a randomized control trial. Omega 2012;65:139‐50.
Kibreab G. Displacement, host governments ‘policies and constraints on the construction of sustainable livelihoods. International Social Science Journal 2003;55:57‐67.
Kibreab G. Pulling the wool over the eyes of the strangers: Refugee deceit and trickery in institutionalized settings. Journal of Refugee Studies 2004;17:1‐28.
Kinzie JD, Kinzie JM, Sedighi B, Woticha A, Mohamed H, Riley C. Prospective one‐year treatment outcomes of tortured refugees: a psychiatric approach. Torture 2012;22:1‐10.
Kinzie JD, Leung P. Clonidine in Cambodian patients with posttraumatic stress disorder. The Journal of Nervous and Mental Disease 1989;177(9):546‐50.
Kinzie JD, Sack RL, Riley CM. The polysomnographic effects of clonidine on sleep disorders in posttraumatic stress disorder: a pilot study with Cambodian patients. Journal of Nervous and Mental Disease 1994;182(10):585‐87.
Kruse J, Joksimovic L, Cavka M, Woller W, Schmitz N. Effects of trauma‐focused psychotherapy upon war refugees. Journal of Traumatic Stress 2009;22:585‐92.
Lange‐Nielsen II, Kolltveit S, Thabet AAM, Pallesen ADS, Johnsen TB, Laberg JC. Short‐term effects of writing intervention among adolescents in Gaza. Journal of Loss and Trauma 2012;17:403‐22.
Lehnung M, Shapiro E, Schreiber M, Hofmann A. Evaluating the EMDR group traumatic episode protocol with refugees: a field study. Journal of EMDR Practice and Research 2017;11:129‐38.
Liedl A, Muller J, Morina N, Karl A, Denke C, Knaevelsrud C. Physical activity within a CBT intervention improves coping with pain in traumatized refugees: results of a randomized controlled design. Pain Medicine 2011;12:234–45.
Marshall LW. Toward a new definition of ‘refugee’: is the 1951 convention out of date? European Journal of Trauma and Emergency Surgery 2011;37:61‐66.
Mattoon L. The gift of trauma: stories of posttraumatic growth and spiritual transformation in war survivors from Uganda and Vietnam. Palo Alto (US): Institute of Transpersonal Psychology, 2011.
McDougal L, Beard J. Revisiting Sphere: new standards of service delivery for new trends in protracted displacement. Disasters 2011;35:87‐101.
Meffert SM, Abdo AO, Alla OAA, Elmakki YOM, Omer AAI, Yousif S, et al. A pilot randomized controlled trial of interpersonal psychotherapy for Sudanese refugees in Cairo, Egypt. Psychological Trauma: Theory, Research, Practice, and Policy 2014;6:240‐59.
Miller K, Billings D. Playing to grow: a primary mental health intervention with Guatemalan refugee children. American Journal of Orthopsychiatry 1994;64(3):346‐56.
Miller KE, Rasmussen A. War exposure, daily stressors, and mental health in conflict and post‐conflict settings: bridging the divide between trauma‐focused and psychosocial frameworks. Social Science & Medicine 2010;70:7‐16.
Mirghani Z. Healing through sharing: An outreach project with Iraqi refugee volunteers in Syria. Intervention 2013;11:321‐29.
Möhlen H, Parzer P, Resch F, Brunner R. Psychosocial support for war‐traumatized child and adolescent refugees: Evaluation of a short‐term treatment program. Australian and New Zealand Journal of Psychiatry 2005;39(1‐2):81‐7.
Mollica RF, Wyshak G, Lavelle J, Truong T, Tor S, Yang T. Assessing symptom change in Southeast Asian refugee survivors of mass violence and torture. American Journal of Psychiatry 1990;147:83‐88.
Morath J, Gola H, Sommershof A, Hamuni G, Kolassa S, Catani C, et al. The effect of trauma‐focused therapy on the altered T cell distribution in individuals with PTSD: evidence from a randomized controlled trial. Journal of Psychiatric Research 2014;54:1‐10.
Moulin C. Border languages: rumors and (dis)placements of (inter)national politics. Alternatives 2010;35:347‐71.
Mowafi H. Conflict, displacement and health in the Middle East. Global Public Health 2011;6:472‐87.
Muller J, Karl A, Denke C, Mathier F, Dittmann J, Rohleder N, et al. Biofeedback for pain management in traumatized refugees. Cognitive Behavioural Therapy 2009;38:184‐90.
Neuner F, Kurreck S, Ruf M, Odenwald M, Elbert T, Schauer M. Can asylum‐seekers with posttraumatic stress disorder be successfully treated? A randomized controlled pilot study. Cognitive Behaviour Therapy 2010;39:81‐91.
Neuner F, Onyut PL, Ertl V, Odenwald M, Schauer E, Elbert T. Treatment of posttraumatic stress disorder by trained lay counselors in an African refugee settlement: a randomized controlled trial. Journal of Consulting and Clinical Psychology 2008;76:686‐94.
Neuner F, Schauer M, Klaschik C, Elbert T. A comparison of narrative exposure therapy, supportive counseling, and psychoeducation for treating posttraumatic stress disorder in an African refugee settlement. Journal of Consulting and Clinical Psychology 2004;72:579‐87.
Neuner F, Schauer M, Roth WT, Elbert T. A narrative exposure treatment as intervention in a refugee camp: a case report. Behavioral Cognitive Psychotherapy 2002;30(2):205‐9.
O’Shea B, Hodes M, Down G, Bramley J. A school‐based mental health service for refugee children. Clinical Child Psychology and Psychiatry 2000;5(2),189‐201.
Onyut LP, Neuner F, Schauer E, Ertl V, Odenwald M, Schauer M, et al. Narrative Exposure Therapy as a treatment for child war survivors with posttraumatic stress disorder: Two case reports and a pilot study in an African refugee settlement. BMC Psychiatry 2005;5:7.
Onyut LP, Neuner F, Schauer E, Ertl V, Odenwald M, Schauer M, et al. The Nakivale Camp Mental Health Project: building local competency for psychological assistance to traumatised refugees. Intervention: International Journal of Mental Health, Psychosocial Work and Counselling in Areas of Armed Conflict. 2004;2(2):90‐107.
Ooi CS, Rooney RM, Roberts C, Kane RT, Wright B, Chatzisarantis N. The efficacy of a group cognitive behavioral therapy for war‐affected young migrants living in Australia: a cluster randomized controlled trial. Frontiers in Psychology 2016;7:1‐14.
Ooi CS. The efficacy and social validity of a group cognitive behavioural therapy for young migrants from war‐affected countries [PhD thesis]. Perth (Australia): Curtin University, 2012.
Oras R, de Ezpeleta SC, Ahmad A. Treatment of traumatized refugee children with Eye Movement Desensitization and Reprocessing in a psychodynamic context. Nordic Journal of Psychiatry 2004;58(3):199‐203.
Otto MW, Hinton D, Korbly NB, Chea A, Ba P, Gershuny BS, et al. Treatment of pharmacotherapy‐refractory posttraumatic stress disorder among Cambodian refugees: a pilot study of combination treatment with cognitive‐behavior therapy v. sertraline alone. Behaviour Research and Therapy 2003;41:1271‐276.
Palic S, Elklit A. An explorative outcome study of CBT‐based multidisciplinary treatment in a diverse group of refugees from a Danish treatment centre for rehabilitation of traumatized refugees. Torture 2009;19:248‐70.
Palmgren PA. Irregular networks: Bangkok refugees in the city and region. Journal of Refugee Studies 2013;27:21‐41.
Paunovic N, Öst LG. Cognitive‐behavior therapy vs. exposure therapy in the treatment of PTSD in refugees. Behaviour Research and Therapy 2001;39:1183‐197.
Pfeiffer E, Goldbeck L. Evaluation of a trauma‐focused group intervention for unaccompanied young refugees: a pilot study. Journal of Traumatic Stress 2017;30(5):531‐36.
Pishyar R. Using de‐reflection techniques to affect clinical change in depression in an Afghan refugee community. International Forum of Logotherapy 2012;35:85‐90.
Porter M, Haslam N. Predisplacement and postdisplacement factors associated with mental health of refugees and internally displaced persons: a meta‐analysis. JAMA 2005; 294: 602‐12.
Quosh C, Eloul L, Ajlani R. Mental health of refugees and displaced persons in Syria and surrounding countries: a systematic review. Intervention 2013;11:276‐94.
Raghavan S, Rasmussen A, Rosenfeld B, Keller AS. Correlates of symptom reduction in treatment‐seeking survivors of torture. Psychological Trauma: Theory, Research, Practice, and Policy 2013;5:377‐83.
Ramadan A. In the ruins of Nahr AlBarid: understanding the meaning of the camp. Journal of Palestine Studies 2014;40:49‐62.
Ramaliu A, Thurston W. Identifying best practices of community participation in providing services to refugee survivors of torture: a case description. Journal of Immigrant Health 2003;5(4):165‐72.
Rangkla P. Refuge and emplacement through Buddhism: Karen refugees and religious practices in a northwestern border town of Thailand. Asia Pacific Journal of Anthropology 2013;14:8‐22.
Rees B, Travis F, Shapiro D, Chant R. Reduction in posttraumatic stress symptoms in Congolese refugees practicing transcendental meditation. Journal of Traumatic Stress 2013;26:295‐98.
Rees B, Travis F, Shapiro D, Chant R. Significant reductions in posttraumatic stress symptoms in Congolese refugees within 10 days of transcendental meditation practice. Journal of Trauma Stress 2014;27:112‐15.
Renner W, Banninger‐Huber E, Peltzer K. Culture‐Sensitive and Resource Oriented Peer (CROP) – groups as a community‐based intervention for trauma survivors: a randomized controlled pilot study with refugees and asylum seekers from Chechnya. Australasian Journal of Disaster Trauma Studies 2011:1‐13.
Renner W. The effectiveness of psychotherapy with refugees and asylum seekers: preliminary results from an Austrian study. Journal of Immigrant and Minor Health 2009;11:41‐45.
Rousseau C, Benoit M, Gauthier MF, Lacroix L, Alain N, Rojas MV, et al. Classroom drama therapy program for immigrant and refugee adolescents: a pilot study. Clinical Child Psychology and Psychiatry 2007;12(3): 451‐65.
Rousseau C, Benoit M, Lacroix L, Gauthier M. Evaluation of a sandplay program for preschoolers in a multiethnic neighborhood. Journal of Child Psychology and Psychiatry 2009;50:743‐50.
Rousseau C, Drapeau A, Lacrois L, Bagilishya D, Heusch N. Evaluation of a classroom program of creative expression workshops for refugee and immigrant children. Journal of Child Psychology and Psychiatry 2005;46:180‐85.
Ruf M, Schauer M, Neuner F, Catani C, Schauer E, Elbert T. Narrative exposure therapy for 7‐ to 16‐year‐olds: a randomized controlled trial with traumatized refugee children. Journal of Traumatic Stress 2010;23:437‐45.
Schaal S, Elbert T, Neuner F. Narrative exposure therapy versus interpersonal psychotherapy. A pilot randomized controlled trial with Rwandan genocide orphans. Psychotherapy and Psychosomatics 2009;78(5):298‐306.
Schauer M. Trauma treatment for children in war: build‐up of an evidence‐based large‐scale mental health intervention in North‐Eastern Sri Lanka [PhD thesis]. Konstanz (Germany): University of Konstanz, 2008.
Schottelkorb AA, Doumas DM, Garcia R. Treatment for childhood refugee trauma: a randomised trial. International Journal of Play Therapy 2012;21:57‐73.
Schulz PM, Huber LC, Resick PA. Practical applications of cognitive processing therapy with Bosnian refugees: implications for adapting practice to a multicultural clientele. Cognitive and Behavioral Practice 2006;13:310‐21.
Schulz PM, Resick PA, Huber LC, Griffin MG. The effectiveness of cognitive processing therapy for PTSD with refugees in a community setting. Cognitive and Behavioral Practice 2006;13(4): 322‐31.
Schwarz‐Langer G, Deighton RR, Jerg‐Bretzke L, Weisker I, Traue HC. Psychiatric treatment for extremely traumatized civil war refugees from former Yugoslavia. Possibilities and limitations of integrating psychotherapy and medication. Torture 2006;16(2):69‐80.
Šehović, M. Evaluation of results of cognitive behavioural therapy with traumatised children of displaced persons. In: Powell S,Durakovic‐Belko E, editors. The psychosocial consequences of war: results of empirical research from the territory of former Yugoslavia. Sarajevo (Bosnia Herzegovina): UNICEF, 2002;265‐66.
Šestan, D. Participation in a program of psychosocial support and reduction of posttraumatic symptoms in preschool children and their mothers. In: Powell S,Durakovic‐Belko E, editors. The psychosocial consequences of war: results of empirical research from the territory of former Yugoslavia. Sarajevo (Bosnia Herzegovina): UNICEF, 2002;255‐57.
Silove D. The challenges facing mental health programs for post‐conflict and refugee communities. Prehospital Disaster Medicine 2004;19(1):90‐96.
Smajkic A, Weine S, Djuric‐Bijedic Z, Boskailo E, Lewis J, Pavkovic I. Sertraline, paroxetine and venlafaxine in refugee posttraumatic stress disorder with depression symptoms. Journal of Traumatic Stress 2001;14:445‐52.
Snodgrass LL, Yamamoto J, Fredrick C, Ton‐That N, Foy DW, Chan L, et al. Vietnamese refugees with PTSD symptomatology: intervention via a coping skills model. Journal of Traumatic Stress 1993;6:569‐75.
Stenmark H, Catani C, Neuner F, Elbert T, Holen A. Treating PTSD in refugees and asylum seekers within the general health care system. A randomized controlled multi‐center study. Behaviour Research and Therapy 2013;51:641‐47.
Stepakoff S, Hubbard J, Katoh M, Falk E, Mikulu J, Nkhoma P, et al. Trauma healing in refugee camps in Guinea: a psychosocial program for Liberian and Sierra Leonean survivors of torture and war. American Psychology 2006;61:921‐31.
Summerfield D. A critique of seven assumptions behind psychological trauma programmes in war‐affected areas. Social Science and Medicine 1999;48:1449‐462.
Ter Heide FJJ, Mooren GTM, Kleijn W, de Jongh A, Kleber RJ. EMDR versus stabilisation in traumatised asylum seekers and refugees: results of a pilot study. European Journal of Psychotraumatology 2011;2:1‐12.
Ter Heide FJJ, Mooren GTM, Van de Schoot R, de Jongh A, Kleber RJ. Eye movement desensitisation and reprocessing therapy v. stabilisation as usual for refugees: randomised controlled trial. British Journal of Psychiatry 2016;209:311‐18.
Thabet AA, Vostanis P, Karim K. Group crisis intervention for children during ongoing war conflict. European Child and Adolescent Psychiatry 2005;14(5):262‐69.
Tol W, Komproe I, Susanty D, Jordans M, Macy R, de Jong J. School‐based mental health intervention for children affected by political violence in Indonesia. JAMA 2008;300(6):655‐62.
Tol WA, Komproe IH, Jordans MJD, Vallipuram A, Sipsma H, Sivayokan S, et al. Outcomes and moderators of a preventive school‐based mental health intervention for children affected by war in Sri Lanka: a cluster randomized trial. World Psychiatry 2012;11:114‐22.
Unterhitzenberger J, Eberle‐Sejari R, Rassenhofer M, Sukale T, Rosner R, Goldbeck L. Trauma‐focused cognitive behavioral therapy with unaccompanied refugee minors: a case series. BMC Psychiatry 2015;15(260):10.1186/s12888‐015‐0645‐0.
Unterhitzenberger J, Rosner R. Case report: manualized trauma‐focused cognitive behavioural therapy with an unaccompanied refugee minor girl. European Journal of Psychotraumatology 2016;7:10.3402/ejpt.v7.29246.
Van Aken M. Dancing belonging: Contesting dabkeh in the Jordan Valley, Jordan. Journal of Ethnic and Migration Studies 2006;32:203‐22.
Van Wyk S, Schweitzer R, Brough M, Vromanns L, Murray K. A longitudinal study of mental health in refugees from Burma: the impact of therapeutic interventions. Australian and New Zealand Journal of Psychiatry 2012;46(10):995‐1003.
Vickers B. Cognitive model of maintenance and treatment of post‐traumatic stress disorder applied to children and adolescents. Clinical Child Psychology and Psychiatry 2005;10(2):217‐34.
Warner FR. Social support and distress among Q’eqchi’ refugee women in Maya Tecun, Mexico. Medical Anthropology Quarterly 2007;21:193‐217.
Weine S, Kulauzovic Y, Klebic A, Besic S, Mujagic A, Muzurovic, et al. Evaluating a multiple‐family group access intervention for refugees with PTSD. Journal of Marital and Family Therapy 2008;34:149‐64.
Weine SM, Dzubur Kulenovic A, Pavkovic I, Gibbons RD. Testimony psychotherapy in Bosnian refugees: A pilot study. American Journal of Psychiatry 1998;155:1720‐726.
Weine SM, Raina D, Zhubi M, Delesi M, Huseni D, Feetham S, et al. The TAFES multi‐family group intervention for Kosovar refugee: a feasibility study. Journal of Nervous and Mental Disease 2003;191(2):100‐7.
Weinstein N, Khabbaz F, Legate N. Enhancing need satisfaction to reduce psychological distress in Syrian refugees. Journal of Consulting and Clinical Psychology 2016;84:645‐50.
Ying S. A comparative study on psychological counseling to solve immigration psychological problems. Chinese Public Health 2006;22(2):140‐41.
Zaman T. Jockeying for position in the humanitarian field: Iraqi refugees and faith‐based organisations in Damascus. Disasters 2012;36:S126‐S148.
Zetter R. More labels, fewer refugees: Remaking the refugee label in an era of globalization. Journal of Refugee Studies 2007;20:172‐92.

## Differences between protocol and review

We did not search Ovid Global Health and BIREME/PAHO LAtin America and the Caribbean DAtabase (LILACS) because we did not have the access and resources required to do so.

For the NIHR Journals Library, PROSPERO, and Epistemonikos abstracts could not be downloaded and imported in to Covidence. Results from these searches were screened by one reviewer (EU) on the website and, if relevant, records were added to Covidence for full‐text screening in duplicate.

We had planned to create a matrix of primary studies from all included reviews, to show overlap between reviews and identify unique primary studies included in the reviews. However, because of the large number of studies identified in the included reviews, a matrix type figure would have been too large and not informative for this overview. Instead, we reported the total number of records identified and the number of unique records.

In the results and discussion sections of this review, we added a paragraph highlighting the characteristics of two included reviews rated 'high quality' in response to a peer‐reviewer suggestion. Although we had not planned to highlight high‐quality reviews, including this information meant we could point out which reviews might be considered more informative to future research on this topic.

## References

[CD013458-bib-0001] Alfadhli K, Drury J. Psychosocial support among refugees of conflict in developing countries: a critical literature review. Journal of Mental Health and Psychosocial Support in Conflict Affected Areas 2016;14(2):128-41.

[CD013458-bib-0002] Alzaghoul A, McKinlay A. Effectiveness of psychological interventions for treating post-traumatic stress disorder in children and adolescents affected by war, conflicts and man-made disasters in low/middle income countries in the Middle East. A systematic review of randomized controlled trials. crd.york.ac.uk/prospero/display_record.php?RecordID=140370 (accessed 09 September 2019).

[CD013458-bib-0003] Aslam RW, Dowrick C, Hagan R, Waqas A, Winrow EW, Edwards R. Barriers and facilitators to uptake of psychosocial interventions delivered by lay therapists to improve mental health and wellbeing of asylum seekers and migrants: a systematic review. crd.york.ac.uk/prospero/display_record.php?RecordID=104453 (accessed 10 September 2019).

[CD013458-bib-0004] Crumlish N, O'Rourke K. A systematic review of treatments for post-traumatic stress disorder among refugees and asylum-seekers. Journal of Nervous and Mental Disease 2010;198(4):237-51. 10.1097/NMD.0b013e3181d61258 20386252

[CD013458-bib-0005] Danmole O, Seguin M, Kousoulis A. Preventative interventions for the mental health of asylum-seekers and refugees in Europe: a systematic review. crd.york.ac.uk/prospero/display_record.php?RecordID=70788 (accessed 10 September 2019).

[CD013458-bib-0006] Eberle-Sejari R, Nocon A, Rosner R. Treatment of posttraumatic symptoms in child and adolescent refugees: a systematic review [German]. Kindheit und Entwicklung 2015;24(3):156-69.

[CD013458-bib-0007] Gwozdziewycz N, Mehl-Madrona L. Meta-analysis of the use of narrative exposure therapy for the effects of trauma among refugee populations. Permanente Journal 2013;17(1):70-6. 10.7812/TPP/12-058 PMC3627789 23596375

[CD013458-bib-0008] Hameed Y. Therapeutic interventions for post-traumatic stress disorders (PTSD) in refugees: systematic review and meta-analysis. crd.york.ac.uk/prospero/display_record.php?RecordID=65481 (accessed 10 September 2019).

[CD013458-bib-0009] Jaroudy S, Thornicroft G, Molyneaux E. Are psychosocial interventions effective for reducing symptoms of depression, anxiety or PTSD in displaced or refugee adults in low or middle income countries who have been affected by conflict? A systematic review protocol and planned meta-analysis. crd.york.ac.uk/prospero/display_record.php?RecordID=102081 (accessed 09 September 2019).

[CD013458-bib-0010] Kobayashi A, Bhandari D, Etienne BM, Sakamoto JL, Khadka N, Masuda S, et al. Participatory action research in addressing mental and psychosocial health in displaced people: a systematic review. crd.york.ac.uk/PROSPERO/display_record.php?RecordID=80838 (accessed 14 October 2019).

[CD013458-bib-0011] Lambert JE, Alhassoon OM. Trauma-focused therapy for refugees: meta-analytic findings. Journal of Counseling Psychology 2015;62(1):28-37. 10.1037/cou0000048 25485547

[CD013458-bib-0012] Lawton K. Does cognitive behavioural therapy (CBT) reduce symptoms of psychological distress and mental illness in children and adolescent refugees and asylum seekers? crd.york.ac.uk/prospero/display_record.php?RecordID=121469 (accessed 09 September 2019).

[CD013458-bib-0013] Lindert J, Von Ehrenstein O, Bain P, Mollica R. Efficiency of mental health interventions for refugees. crd.york.ac.uk/prospero/display_record.php?RecordID=33802 (accessed 09 September 2019).

[CD013458-bib-0014] Liu Q, Wang H, Wang W, Zhang F, Li J, Song WF, et al. Interventions on preventing and treating mental health problems of involuntary migrants: a systematic review. Chinese Journal of Evidence-Based Medicine 2009;9(9):929-37.

[CD013458-bib-0015] Meinhart M, Mills E, Michalopoulos L, El-Bassal N. Systematic review of Syrian refugee mental health response, intervention and analyses. crd.york.ac.uk/PROSPERO/display_record.php?RecordID=51923 (accessed 14 October 2019).

[CD013458-bib-0016] Mitra R, Hodes M. Prevention of psychological distress and promotion of resilience amongst unaccompanied refugee minors in resettlement countries. Child: Care, Health and Development 2019;45(2):198-215. 10.1111/cch.12640 30661259

[CD013458-bib-0017] Miyazaki C, Suto M, Kataoka C. Systematic review and network meta-analysis of low-intensity psychological interventions for improving mental health in displaced children. crd.york.ac.uk/prospero/display_record.php?RecordID=83959 (accessed 09 September 2019).

[CD013458-bib-0018] Morina N, Sterr TN. Lack of evidence for the efficacy of psychotherapies for PTSD and depression in child and adolescent refugees. World Psychiatry 2019;18(1):107-8. 10.1002/wps.20596 PMC6313233 30600622

[CD013458-bib-0019] Nakeyar C, Frewen PA. Evidence-based care for Iraqi, Kurdish, and Syrian asylum seekers and refugees of the Syrian civil war: a systematic review. Canadian Psychology 2016;57(4):233-45.

[CD013458-bib-0020] Naseh M, Macgowan MJ, Wagner EF, Zahra A, Miriam P, Stuart PH. Cultural adaptations in psychosocial interventions for post-traumatic stress disorder among refugees: a systematic review. Journal of Ethnic & Cultural Diversity in Social Work 2019;28(1):76-97.

[CD013458-bib-0021] Nickerson A, Bryant RA, Silove D, Steel Z. A critical review of psychological treatments of posttraumatic stress disorder in refugees. Clinical Psychology Review 2011;31(3):399-417. 10.1016/j.cpr.2010.10.004 21112681

[CD013458-bib-0022] Nocon A, Eberle-Sejari R, Unterhitzenberger J, Rosner R. The effectiveness of psychosocial interventions in war-traumatized refugee and internally displaced minors: systematic review and meta-analysis. European Journal of Psychotraumatology 2017;8(Suppl 2):1-15. 10.1080/20008198.2017.1388709 PMC5687794 29163868

[CD013458-bib-0023] Nosè M, Ballette F, Turrini G, Purgato M, Barbui C. Effectiveness of narrative exposure therapy in refugees and asylum seekers with trauma-spectrum disorders: a systematic review. crd.york.ac.uk/prospero/display_record.php?RecordID=50377 (accessed 09 September 2019).

[CD013458-bib-0024] Nosè M, Ballette F, Bighelli I, Turrini G, Purgato M, Tol W, et al. Psychosocial interventions for post-traumatic stress disorder in refugees and asylum seekers resettled in high-income countries: systematic review and meta-analysis. PLOS One 2017;12(2):e0171030. 10.1371/journal.pone.0171030 PMC5289495 28151992

[CD013458-bib-0025] Palic S, Elklit A. Psychosocial treatment of posttraumatic stress disorder in adult refugees: a systematic review of prospective treatment outcome studies and a critique. Journal of Affective Disorders 2011;131(1-3):8-23. 10.1016/j.jad.2010.07.005 20708804

[CD013458-bib-0026] Phillips K, Bradfield E, Hogan S, Sheffield D, Baker C. Art therapy and participatory art for the well-being of refugees: a systematic review. crd.york.ac.uk/prospero/display_record.php?RecordID=59967 (accessed 09 September 2019).

[CD013458-bib-0027] Sims K, Pooley JA. Posttraumatic growth amongst refugee populations: a systematic review. In: The Routledge International Handbook of Psychosocial Resilience. London: Routledge, 2017:230-47.

[CD013458-bib-0028] Soltan F, Vanderbloemen L. Systematic review of community-based interventions for improving health and well-being in refugee children and adolescents after resettlement in developed countries. crd.york.ac.uk/prospero/display_record.php?RecordID=99102 (accessed 09 September 2019).

[CD013458-bib-0029] Sonne C, Carlsson J, Bech P, Mortensen EL. Pharmacological treatment of refugees with trauma-related disorders: what do we know today? Transcultural Psychiatry 2017;54(2):260-80. 10.1177/1363461516682180 27956478

[CD013458-bib-0030] Thompson CT, Vidgen A, Roberts NP. Psychological interventions for post-traumatic stress disorder in refugees and asylum seekers: a systematic review and meta-analysis. Clinical Psychology Review 2018;63:66-79. 10.1016/j.cpr.2018.06.006 29936342

[CD013458-bib-0031] Tribe RH, Sendt KV, Tracy DK. A systematic review of psychosocial interventions for adult refugees and asylum seekers. Journal of Mental Health 2019;28(6):662-76. 10.1080/09638237.2017.1322182 28485636

[CD013458-bib-0032] Turrini G, Purgato M, Ballette F, Nosè M, Ostuzzi G, Barbui C. Common mental disorders in asylum seekers and refugees: umbrella review of prevalence and intervention studies. International Journal of Mental Health Systems 2017;11:51. 10.1186/s13033-017-0156-0 PMC5571637 28855963

[CD013458-bib-0033] Turrini G, Purgato M, Acarturk C, Anttila M, Au T, Ballette F, et al. Efficacy and acceptability of psychosocial interventions in asylum seekers and refugees: systematic review and meta-analysis. Epidemiology and Psychiatric Sciences 2019;28(4):376-88. 10.1017/S2045796019000027 PMC6669989 30739625

[CD013458-bib-0034] Turrini G, Purgato M, Nosè M, Ostuzzi G, Tedeschi F, Barbui C. A network meta-analysis of psychological and psychosocial interventions for refugees and asylum seekers with PTSD. crd.york.ac.uk/prospero/display_record.php?RecordID=126604 (accessed 09 September 2019).

[CD013458-bib-0035] Tyrer RA, Fazel M. School and community-based interventions for refugee and asylum seeking children: a systematic review. PLOS One 2014;9(2):e89359. 10.1371/journal.pone.0089359 PMC3933416 24586715

[CD013458-bib-0036] Van Wyk S, Schweitzer RD. A systematic review of naturalistic interventions in refugee populations. Journal of Immigrant and Minority Health 2014;16(5):968-77. 10.1007/s10903-013-9835-3 23666201

[CD013458-bib-0037] Williams ME, Thompson SC. The use of community-based interventions in reducing morbidity from the psychological impact of conflict-related trauma among refugee populations: a systematic review of the literature. Journal of Immigrant and Minority Health 2011;13(4):780-94. 10.1007/s10903-010-9417-6 21103930

[CD013458-bib-0038] Wright A, Cullen B. Efficacy and cultural adaptations of narrative exposure therapy for treating trauma with refugees/ asylum seekers: a systematic review. crd.york.ac.uk/prospero/display_record.php?RecordID=121055 (accessed 09 September 2019).

[CD013458-bib-0039] Albane B, Lisé C. Education in emergencies in humanitarian responses, results of creative or artistic educational practices in conflict areas and in camps promoting the well-being of children: protocol for a systematic review (meta-synthesis). crd.york.ac.uk/prospero/display_record.php?RecordID=124913 (accessed 09 September 2019).

[CD013458-bib-0040] Aly A. Transdiagnostic cognitive behavioural therapy-based psychological interventions in trauma-exposed adult populations from low- and middle-income countries: a systematic review. crd.york.ac.uk/prospero/display_record.php?RecordID=54897 (accessed 09 September 2019).

[CD013458-bib-0041] Demazure G, Gaultier S, Pinsault N. Dealing with difference: a scoping review of psychotherapeutic interventions with unaccompanied refugee minors. European Child & Adolescent Psychiatry 2018;27(4):447-66. 10.1007/s00787-017-1083-y 29214387

[CD013458-bib-0042] Esala JJ, Vukovich MM, Hanbury A, Kashyap S, Joscelyne A. Collaborative care for refugees and torture survivors: key findings from the literature. Traumatology 2018;24(3):168-85.

[CD013458-bib-0043] Hassan A, Sharif K. Efficacy of telepsychiatry in refugee populations: a systematic review of the evidence. Cureus 2019;11(1):e3984. 10.7759/cureus.3984 PMC6443105 30972263

[CD013458-bib-0044] Ho R, Clark A, Saunders S. What are the facilitators and barriers to access mental health services for refugees and asylum seekers: a systematic review. crd.york.ac.uk/prospero/display_record.php?RecordID=88629 (accessed 10 September 2019).

[CD013458-bib-0045] Koesters M, Barbui C, Purgato M. Recent approaches to provision of mental healthcare in refugee populations. Current Opinion in Psychiatry 2018;31(4):368-72. 10.1097/YCO.0000000000000428 29708893

[CD013458-bib-0046] Liem A, Natari RF, Jimmy J, Hall B. Artificial intelligence applications in mental health care for refugees and immigrants: a rapid review. crd.york.ac.uk/prospero/display_record.php?RecordID=127337 (accessed 09 September 2019).

[CD013458-bib-0047] Logan A, Compean E, Jones J, Hamner M, Kirby C. Asylum seekers, refugees, and immigrants: a systematic review of prevalence, diagnostic, and treatment challenges in PTSD and co-morbid psychosis. crd.york.ac.uk/PROSPERO/display_record.php?RecordID=113048 (accessed 14 October 2019).

[CD013458-bib-0048] Murray KE, Davidson GR, Schweitzer RD. Review of refugee mental health interventions following resettlement: best practices and recommendations. American Journal of Orthopsychiatry 2010;80(4):576-85. 10.1111/j.1939-0025.2010.01062.x PMC3727171 20950298

[CD013458-bib-0049] Nicholl C, Thompson A. The psychological treatment of Post Traumatic Stress Disorder (PTSD) in adult refugees: a review of the current state of psychological therapies. Journal of Mental Health 2004;13(4):351-62.

[CD013458-bib-0050] Purgato M, Del Piccolo L, Rimondini M, Michengigh G, Rudi D, Vitali F, et al. Physical activity interventions for improving mental health outcomes in refugees and asylum seekers: a systematic review and meta-analysis. crd.york.ac.uk/prospero/display_record.php?RecordID=123550 (accessed 10 September 2019).

[CD013458-bib-0051] Quosh C, Eloul L, Ajlani R. Mental health of refugees and displaced persons in Syria and surrounding countries: a systematic review. International Journal of Mental Health, Psychosocial Work & Counselling in Areas of Armed Conflict 2013;11(3):276-94.

[CD013458-bib-0052] Sijbrandij M. Expanding the evidence: key priorities for research on mental health interventions for refugees in high-income countries. Epidemiology and Psychiatric Sciences 2018;27(2):105-8. 10.1017/S2045796017000713 PMC6998957 29143713

[CD013458-bib-0053] Slobodin O, De Jong JT. Family interventions in traumatized immigrants and refugees: a systematic review. International Journal of Social Psychiatry 2015;52(6):723-42. 10.1177/1363461515588855 26047828

[CD013458-bib-0054] Slobodin O, De Jong JT. Mental health interventions for traumatized asylum seekers and refugees: what do we know about their efficacy? Transcultural Psychiatry 2015;61(1):17-26. 10.1177/0020764014535752 24869847

[CD013458-bib-0055] Sullivan AL, Simonson GR. A systematic review of school-based social-emotional interventions for refugee and war-traumatized youth. Review of Educational Research 2016;86(2):503-30.

[CD013458-bib-0056] Wood BM, Kallestrup P. A review of non-specialised, group-based mental health and psychosocial interventions in displaced populations. International Journal of Migration, Health & Social Care 2018;14(3):347-59.

[CD013458-bib-0057] Alemi Q, James S, Cruz R, Zepeda V, Racadio M. Psychological distress in Afghan refugees: a mixed-method systematic review. Journal of Immigrant and Minority Health/Center for Minority Public Health 2014;16(6):1247-61. 10.1007/s10903-013-9861-1 PMC3912229 23784146

[CD013458-bib-0058] Anders M, Christiansen H. Unaccompanied refugee minors: a systematic review of psychological interventions. Kindheit und Entwicklung 2016;25(4):216-30.

[CD013458-bib-0059] American Psychiatric Association. Diagnostic and Statistical Manual of Mental Disorders (DSM-5). 5th edition. Washington DC: American Psychiatric Association, 2013.

[CD013458-bib-0060] Baker FA, Metcalf O, Varker T, O'Donnell M. A systematic review of the efficacy of creative arts therapies in the treatment of adults with PTSD. Psychological Trauma: Theory, Research, Practice and Policy 2018;10(6):643-51. 10.1037/tra0000353 29199839

[CD013458-bib-0061] Barbui C, Purgato M, Churchill R, Adams C, Amato L, Macdonald G, et al. Cochrane for global mental health. Lancet Psychiatry 2017;4(4):e6. 10.1016/S2215-0366(17)30090-1 28347436

[CD013458-bib-0062] Beck AT, Rush AJ, Shaw BF, Emery G. Cognitive Therapy of Depression. New York: Guilford Press, 1979.

[CD013458-bib-0063] Campbell Collaboration. Making an evidence and gap map (EGM). campbellcollaboration.org/explore/making-an-evidence-and-gap-map-egm.html (accessed 12 April 2019).

[CD013458-bib-0064] Cohen JA, Mannarino AP, Deblinger E. Trauma-Focused CBT for Children and Adolescents: Treatment Applications. New York: Guilford Press, 2012.

[CD013458-bib-0065] Covidence. Version accessed 15 May 2020. Melbourne, Australia: Veritas Health Innovation. Available at covidence.org.

[CD013458-bib-0066] Dawson KS, Bryant RA, Harper M, Tay AK, Rahman A, Schafer A, et al. Problem Management Plus (PM+): a WHO transdiagnostic psychological intervention for common mental health problems. World Psychiatry 2015;14(3):354-7. 10.1002/wps.20255 PMC4592660 26407793

[CD013458-bib-0067] Fassil Y, Burnett A. Commissioning mental health services for vulnerable adult migrants. mind.org.uk/media/3168649/vulnerable-migrants_2015_mindweb.pdf (accessed 12 March 2020).

[CD013458-bib-0068] Fazel M, Wheeler J, Danesh J. Prevalence of serious mental disorder in 7000 refugees resettled in western countries: a systematic review. Lancet 2005;365(9467):1309-14. 10.1016/S0140-6736(05)61027-6 15823380

[CD013458-bib-0069] Feighner JP. Mechanism of action of antidepressant medications. Journal of Clinical Psychiatry 1999;60(Suppl 4):4-11. 10086478

[CD013458-bib-0070] Foa EB, McLean CP. The efficacy of exposure therapy for anxiety-related disorders and its underlying mechanisms: the case of OCD and PTSD. Annual Review of Clinical Psychology 2016;12:1-28. 10.1146/annurev-clinpsy-021815-093533 26565122

[CD013458-bib-0071] Freud S. An Outline of Psychoanalysis. London: Hogarth Press, 1949.

[CD013458-bib-0072] Higgins JP, Green S (editors). Cochrane Handbook for Systematic Reviews of Interventions Version 5.1.0 (updated March 2011). The Cochrane Collaboration, 2011. Available from handbook.cochrane.org.

[CD013458-bib-0073] Hofmann SG, Asmundson GJG. Acceptance and mindfulness-based therapy: new wave or old hat? Clinical Psychology Review 2008;28(1):1-16. 10.1016/j.cpr.2007.09.003 17904260

[CD013458-bib-0074] Jackson HJ, Moss JD, Solinski S. Social skills training - an effective treatment for unipolar nonpsychotic depression. Australian and New Zealand Journal of Psychiatry 1985;19(4):342-53. 10.1080/00048678509158842 3914279

[CD013458-bib-0075] Kanter JW, Ajeng JP, Maria MS, Gabriela AN. Behavioural activation: history, evidence and promise. British Journal of Psychiatry 2012;200(5):361-3. 10.1192/bjp.bp.111.103390 PMC13473189 22550329

[CD013458-bib-0076] Kendrick T, Pilling S. Common mental health disorders: identification and pathways to care: NICE clinical guideline. British Journal of General Practice 2012;62(594):47-9. 10.3399/bjgp12X616481 PMC3252532 22520681

[CD013458-bib-0077] Khamis V. Posttraumatic stress disorder and emotion dysregulation among Syrian refugee children and adolescents resettled in Lebanon and Jordan. Child Abuse & Neglect 2019;89:29-39. 10.1016/j.chiabu.2018.12.013 30612072

[CD013458-bib-0078] Khan K, Krafft T, Diaz E, Ledoux C. Evidence on interventions that improve mental health of child refugees and child asylum seekers in Europe: a rapid systematic review. European Journal of Public Health 2018;28:106.

[CD013458-bib-0079] Landin-Romero R, Moreno-Alcazar A, Pagani M, Amann BL. How does eye movement desensitization and reprocessing therapy work? A systematic review on suggested mechanisms of action. Frontiers in Psychology 2018;9:1395. 10.3389/fpsyg.2018.01395 PMC6106867 30166975

[CD013458-bib-0080] Lindert J, Von Ehrenstein OS, Priebe S, Mielck A, Brähler E. Depression and anxiety in labor migrants and refugees: a systematic review and meta-analysis. Social Science & Medicine 2009;69(2):246-57. 10.1016/j.socscimed.2009.04.032 19539414

[CD013458-bib-0081] Mansell W, Harvey A, Watkins E, Shafran R. Conceptual foundations of the transdiagnostic approach to CBT. Journal of Cognitive Psychotherapy 2009;23(1):6-19.

[CD013458-bib-0082] McDonald A, Save the Children. Invisible wounds. savethechildren.org.uk/content/dam/global/reports/emergency-humanitarian-response/invisible-wounds.pdf (accessed 12 March 2020).

[CD013458-bib-0083] McEvoy PM, Nathan P, Norton PJ. Efficacy of transdiagnostic treatments: a review of published outcome studies and future research directions. Journal of Cognitive Psychotherapy 2009;23(1):20-33.

[CD013458-bib-0084] Miake-Lye IM, Hempel S, Shanman R, Shekelle PG. What is an evidence map? A systematic review of published evidence maps and their definitions, methods, and products. Systematic Reviews 2016;5:28. 10.1186/s13643-016-0204-x PMC4750281 26864942

[CD013458-bib-0085] Moher D, Liberati A, Tetzlaff J, Altman DG. Preferred reporting items for systematic reviews and meta-analyses: the PRISMA statement. Annals of Internal Medicine 2009;151(4):264-9. 10.7326/0003-4819-151-4-200908180-00135 19622511

[CD013458-bib-0086] Murray LK, Dorsey S, Haroz E, Lee C, Alsiary MM, Haydary A, et al. A common elements treatment approach for adult mental health problems in low- and middle-income countries. Cognitive and Behavioral Practice 2015;21(2):111-23. 10.1016/j.cbpra.2013.06.005 PMC4304666 25620867

[CD013458-bib-0087] Neuner F, Onyut PL, Ertl V, Odenwald M, Schauer E, Elbert T. Treatment of posttraumatic stress disorder by trained lay counselors in an African refugee settlement: a randomized controlled trial. Journal of Consulting and Clinical Psychology 2008;76:686-94. 10.1037/0022-006X.76.4.686 18665696

[CD013458-bib-0088] Neuner F, Kurreck S, Ruf M, Odenwald M, Elbert T, Schauer M. Can asylum-seekers with posttraumatic stress disorder be successfully treated? A randomized controlled pilot study. Cognitive Behaviour Therapy 2010;39:81-91. 10.1080/16506070903121042 19816834

[CD013458-bib-0089] NICE. NICE guideline [NG116]: Post-traumatic stress disorder. nice.org.uk/guidance/ng116 (accessed 12 March 2020).

[CD013458-bib-0090] NICE. NICE guideline [NG134]: Depression in children and young people: identification and management. nice.org.uk/guidance/ng134 (accessed 12 March 2020).

[CD013458-bib-0091] O'Leary BC, Woodcock P, Kaiser MJ, Pullin AS. Evidence maps and evidence gaps: evidence review mapping as a method for collating and appraising evidence reviews to inform research and policy. Environmental Evidence 2017;6:19. [DOI: doi.org/10.1186/s13750-017-0096-9]

[CD013458-bib-0092] Piegenschke K, Sihorsch M, Christiansen H. Accompanied refugee minors: a systematic review of psychological interventions with family inclusion. Kindheit und Entwicklung 2019;28(3):147-59.

[CD013458-bib-0093] Ranasighe PD, Levy BR. Prevalence of and sex disparities in posttraumatic stress disorder in an internally displaced Sri Lankan population 6 months after the 2004 tsunami. Disaster Medicine and Public Health Preparedness 2007;1(1):34-41. 10.1097/DMP.0b013e318068fbb7 18388601

[CD013458-bib-0094] Roberts B, Ocaka KF, Browne J, Oyok T, Sondorp E. Factors associated with post-traumatic stress disorder and depression amongst internally displaced persons in northern Uganda. BMC Psychiatry 2008;8(1):38. 10.1186/1471-244X-8-38 PMC2397420 18489768

[CD013458-bib-0095] Robjant K, Fazel M. The emerging evidence for Narrative Exposure Therapy: a review. Clinical Psychology Review 2010;30:1030-9. 10.1016/j.cpr.2010.07.004 20832922

[CD013458-bib-0096] Rogers C. Client-Centered Therapy: Its Current Practice, Implications and Theory. London: Constable, 1951.

[CD013458-bib-0097] Rose SC, Bisson J, Churchill R, Wessely S. Psychological debriefing for preventing post traumatic stress disorder (PTSD). Cochrane Database of Systematic Reviews 2002, Issue 2. Art. No: CD000560. [DOI: 10.1002/14651858.CD000560] 12076399

[CD013458-bib-0098] Schauer M, Schauer M, Neuner F, Elbert T. Narrative Exposure Therapy: A Short-Term Treatment for Traumatic Stress Disorders. 2nd (revised) edition. Boston: Hogrefe Publishing, 2011.

[CD013458-bib-0099] Shamseer L, Moher D, Clarke M, Ghersi D, Liberati A, Petticrew M, et al. Preferred reporting items for systematic review and meta-analysis protocols (PRISMA-P) 2015: elaboration and explanation. BMJ 2015;350:g7647. 10.1136/bmj.g7647 25555855

[CD013458-bib-0100] Shapiro F. Eye Movement Desensitization and Reprocessing (EMDR) Therapy: Basic Principles, Protocols, and Procedures. New York: Guilford Publications, 2017.

[CD013458-bib-0101] Sharan P, Gallo C, Gureje O, Lamberte E, Mari JJ, Mazzotti G, et al. Mental health research priorities in low- and middle-income countries of Africa, Asia, Latin America and the Caribbean. British Journal of Psychiatry 2009;195(4):354-63. 10.1192/bjp.bp.108.050187 PMC3432479 19794206

[CD013458-bib-0102] Shea BJ, Reeves BC, Wells G, Thuku M, Hamel C, Moran J, et al. AMSTAR 2: a critical appraisal tool for systematic reviews that include randomised or non-randomised studies of healthcare interventions, or both. BMJ 2017;358:j4008. 10.1136/bmj.j4008 PMC5833365 28935701

[CD013458-bib-0103] Silove D, Ventevogel P, Rees S. The contemporary refugee crisis: an overview of mental health challenges. World Psychiatry 2017;16(2):130-9. 10.1002/wps.20438 PMC5428192 28498581

[CD013458-bib-0104] Stiles WB, Barkham M, Mellor-Clark J, Connell J. Effectiveness of cognitive-behavioural, person-centred, and psychodynamic therapies in UK primary-care routine practice: replication in a larger sample. Psychological Medicine 2008;38(5):677-88. 10.1017/S0033291707001511 17825124

[CD013458-bib-0105] Tol WA, Purgato M, Bass JK, Galappatti A, Eaton W. Mental health and psychosocial support in humanitarian settings: a public mental health perspective. Epidemiology and Psychiatric Sciences 2015;24:484-94. 10.1017/S2045796015000827 PMC8367376 26399635

[CD013458-bib-0106] UNHCR UN Refugee Agency. 1951 Convention on the status of refugees. unhcr.org/asylum-and-migration.html (accessed 1 March 2019).

[CD013458-bib-0107] Wainberg ML, Scorza P, Shultz JM, Helpman L, Mootz JJ, Johnson KA, et al. Challenges and opportunities in global mental health: a research-to-practice perspective. Current Psychiatry Reports 2017;19(5):28. 10.1007/s11920-017-0780-z PMC5553319 28425023

